# Monophyletic Origin and Divergent Evolution of Animal Telomerase RNA

**DOI:** 10.1093/molbev/msaa203

**Published:** 2020-08-08

**Authors:** Dhenugen Logeswaran, Yang Li, Joshua D Podlevsky, Julian J -L Chen

**Affiliations:** School of Molecular Sciences, Arizona State University, Tempe, AZ

**Keywords:** noncoding RNA, homology search, RNA structure, telomere, ribonucleoprotein

## Abstract

Telomerase RNA (TR) is a noncoding RNA essential for the function of telomerase ribonucleoprotein. TRs from vertebrates, fungi, ciliates, and plants exhibit extreme diversity in size, sequence, secondary structure, and biogenesis pathway. However, the evolutionary pathways leading to such unusual diversity among eukaryotic kingdoms remain elusive. Within the metazoan kingdom, the study of TR has been limited to vertebrates and echinoderms. To understand the origin and evolution of TR across the animal kingdom, we employed a phylogeny-guided, structure-based bioinformatics approach to identify 82 novel TRs from eight previously unexplored metazoan phyla, including the basal-branching sponges. Synthetic TRs from two representative species, a hemichordate and a mollusk, reconstitute active telomerase in vitro with their corresponding telomerase reverse transcriptase components, confirming that they are authentic TRs. Comparative analysis shows that three functional domains, template-pseudoknot (T-PK), CR4/5, and box H/ACA, are conserved between vertebrate and the basal metazoan lineages, indicating a monophyletic origin of the animal TRs with a snoRNA-related biogenesis mechanism. Nonetheless, TRs along separate animal lineages evolved with divergent structural elements in the T-PK and CR4/5 domains. For example, TRs from echinoderms and protostomes lack the canonical CR4/5 and have independently evolved functionally equivalent domains with different secondary structures. In the T-PK domain, a P1.1 stem common in most metazoan clades defines the template boundary, which is replaced by a P1-defined boundary in vertebrates. This study provides unprecedented insight into the divergent evolution of detailed TR secondary structures across broad metazoan lineages, revealing ancestral and later-diversified elements.

## Introduction

Telomerase is a ribonucleoprotein (RNP) enzyme that maintains telomere function and genome integrity by processively adding telomeric DNA repeats onto chromosome ends, which is the most common solution to the end-replication problem of linear chromosomes in eukaryotes (Shay and Wright 2019). The core components of telomerase include telomerase reverse transcriptase (TERT) that catalyzes DNA polymerization and the integral telomerase RNA (TR) component that provides the template for telomeric DNA synthesis. In addition to providing the template, TR harbors structural domains that confer telomerase enzymatic activity and serve as a scaffold for binding a variety of accessory proteins (Musgrove et al. 2018). These accessory proteins play important roles in RNP biogenesis and functional regulation of the telomerase enzyme in vivo (Podlevsky and Chen 2016).

TR varies dramatically in size, primary sequence, secondary structure, and biogenesis pathway among different groups of eukaryotes. The sizes of TRs range from 140 to 210 nt in ciliates, 235 to 347 nt in plants, 312 to 559 nt in vertebrates, and 900 to 2425 nt in fungi (Podlevsky and Chen 2016; Song et al. 2019). This dramatic size expansion in vertebrate and fungal TRs results from addition of group-specific RNA structural domains that serve as binding sites for accessory proteins. In addition to size variation, TRs show extremely low sequence similarity even among phylogenetically closely related species, which drastically hinders TR identification in many important model organisms using sequence-based bioinformatics approaches.

Within the animal kingdom, the study of TR secondary structure and function has been limited to vertebrates and echinoderms, leaving the vast majority of the metazoan phyla unexplored (Chen et al. 2000; Xie et al. 2008; Li et al. 2013; Podlevsky et al. 2016b). Within vertebrate TRs, two conserved structural domains are essential for telomerase catalysis. The first one is the template-pseudoknot (T-PK) domain which harbors a single-stranded template region corresponding to 1.5–2 copies of the 6-nt telomeric DNA repeat. The 5′ boundary of the template is physically defined by a template boundary element (TBE) that prevents the flanking sequence from being used as template to incorporate nontelomeric sequence into telomeric DNA (Chen and Greider 2003b). Located downstream of the template is the pseudoknot structure that is essential for TERT-TR interaction and enzyme activity (Blackburn and Collins 2011; Podlevsky and Chen 2012). NMR and biochemical studies of the PK fragment of TR revealed a triple-helix structure that plays an essential role in telomerase function (Chen and Greider 2005; Theimer et al. 2005). The second essential domain within vertebrate TR is called CR4/5 which can reconstitute telomerase activity in trans as a separate RNA fragment together with the T-PK domain (Mitchell and Collins 2000; Chen et al. 2002; Mason et al. 2003; Xie et al. 2008). Interestingly, echinoderm TRs lack the vertebrate CR4/5 and instead possess a domain that has different secondary structure but is functionally equivalent to the CR4/5, thus called eCR4/5. Similarly, the echinoderm eCR4/5 domain can bind independently to echinoderm TERT in trans to promote telomerase activity in vitro (Podlevsky et al. 2016b). This requirement of two TR structural domains for telomerase activity is universally conserved among all major groups of eukaryotes from *Trypanosome* to vertebrates (Podlevsky and Chen 2016).

A third TR structural domain, called box H/ACA, is essential for telomerase RNP processing and biogenesis in vivo (Mitchell et al. 1999). This box H/ACA domain is conserved in both vertebrate and echinoderm TRs, containing structural motifs similar to the box H/ACA small nucleolar RNA (snoRNA) and binds the protein components of the box H/ACA snoRNP, including dyskerin, NOP10, NHP2, and GAR1, which protect the 3′-end of the mature TR from exonuclease degradation (Wang and Meier 2004; Egan and Collins 2010; Nguyen et al. 2018; Tseng et al. 2018). In addition to the dyskerin complex, TRs from most vertebrates, excluding teleost fish (Xie et al. 2008), contain a conserved motif called the CAB box in the distal loop of P8 that binds a protein called TCAB1 which belongs to a subset of snoRNP, called the small Cajal body RNP (scaRNP), and is important for TR localization to the Cajal body in the nucleus (Jády et al. 2004; Venteicher et al. 2009). Similarly, the CAB box motif is not universally conserved in echinoderm TRs (Podlevsky et al. 2016b). It is not clear whether the sno- or scaRNA-related biogenesis of vertebrate TR is conserved throughout the animal kingdom. However, the TRs from other eukaryotic kingdoms utilized surprisingly divergent biogenesis pathways, each of which is shared with other noncoding RNAs—a snRNA-like pathway in yeast TRs (Seto et al. 1999), a LARP7 family protein mediated mechanism in ciliate TR (Witkin and Collins 2004), and a Box C/D snoRNA-like pathway in Trypanosome TR (Gupta et al. 2013). How these distinct structural domains and divergent biogenesis mechanisms originated and evolved along different major eukaryotic lineages remain puzzling.

TR identification in a new group of eukaryotes typically involves biochemical purification of telomerase holoenzyme from cell lysates (Greider and Blackburn 1989; Leonardi et al. 2008; Qi et al. 2013). However, biochemical approaches are often challenging and sometime infeasible due to the low abundance of the telomerase enzyme, the lack of genetic manipulation tools, or the lack of a scalable culture procedure. Polymerase chain reaction (PCR)-based approaches (Chen et al. 2000; Dandjinou et al. 2004; Gunisova et al. 2009), as well as bioinformatics tools such as the Basic Local Alignment Search Tool (BLAST) (Altschul et al. 1990) or Fragrep2 that uses position-specific weight matrices (PWMs) as a search pattern (Mosig et al. 2007), have been successfully employed to identify TRs but limited to closely related species (Xie et al. 2008; Qi et al. 2013; Podlevsky et al. 2016b).

The identification of TR genes in many metazoan species has been challenging and unsuccessful, which limits our understanding of TR evolution across the animal kingdom. Herein, we report the identification of TR sequences from major animal phyla including the early-branching sponges. The comparative analysis of the metazoan TR secondary structures revealed three ancestral structural core domains, pseudoknot, CR4/5, and box H/ACA, that are conserved between the basal-branching sponges and vertebrates, supporting a monophyletic origin of animal TRs and revealing the divergent evolution of TR secondary structures. Interestingly, the CR4/5 domain appears to be relatively more adaptable than other domains and seems diversified twice along two separate lineages with the loss of the crucial P6.1 stem-loop. This study provides the first global glance of TR structure evolution along specific lineages within the animal kingdom.

## Results

### A Phylogeny-Guided Approach for Finding TR Homologs in the Animal Kingdom

**Fig. 1.**
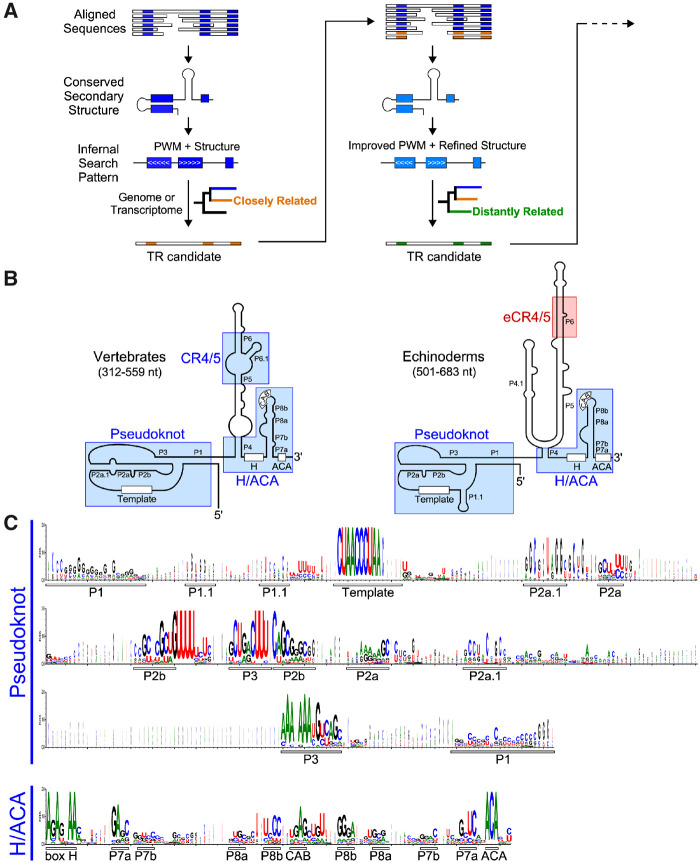
A phylogeny-assisted approach for TR identification. (*A*) The workflow of phylogeny-assisted reiterative homology search for novel TRs. An initial PWM and secondary structure pattern derived from known TRs was used to search for novel TR candidates in the genomes or transcriptomes of closely related species. The newly identified TR sequences were used to improve the PWM and structural pattern for searching TR candidates in distantly related species. (*B*) Comparison of vertebrate and echinoderm TR secondary structures. Two structural domains, T-PK and box H/ACA domains, are conserved in both vertebrate and echinoderm TRs, whereas the vertebrate CR4/5 domain that contains P6.1 stem-loop is replaced with a functionally equivalent eCR4/5 domain in echinoderms. The size ranges of vertebrate and echinoderm TRs are shown. (*C*) PWMs of TR sequences of the pseudoknot and H/ACA domains. PWMs are derived from the multiple-sequence alignment of 42 vertebrate TRs (Chen et al. 2000) and 13 echinoderm TRs (Li et al. 2013; Podlevsky et al. 2016b). Conserved sequence motifs and base-paired regions within each domain are indicated underneath the matrices

We devised a bioinformatics approach to leverage the publicly available genome and transcriptome sequencing data for finding TR homologs in previously unexplored metazoan species and, based on phylogenetic distance, we targeted initially the species phylogenetically closely related to those with TR genes identified (fig. 1*A*). Our search strategy primarily employed the INFERNAL (INFERence of RNA ALignments) program (Nawrocki and Eddy 2013; Barquist et al. 2016) that searches for sequence similarity as well as conserved secondary structure features. For training the INFERNAL program, we used the aligned sequences and conserved secondary structures of known TRs from vertebrates and echinoderms, two major clades in deuterostomes (Chen et al. 2000; Xie et al. 2008; Li et al. 2013; Podlevsky et al. 2016b). Both vertebrate and echinoderm TRs comprise three distinct conserved structural domains, the pseudoknot, CR4/5 (or eCR4/5 for echinoderm), and box H/ACA (fig. 1*B*). As the vertebrate CR4/5 domain is not conserved in the echinoderm TR, we performed INFERNAL analysis using a search pattern derived from the sequence alignment of only pseudoknot and H/ACA domains excluding the CR4/5 domain (fig. 1*C*).

The detailed sequence alignment of vertebrate and echinoderm TRs revealed regions with conservation in nucleotide identity (supplementary fig. S1*A* and S1*B*, Supplementary Material online). For the pseudoknot domain, the most conserved nucleotide residues are in the template as well as helices P2b and P3 that form a pseudoknot structure (fig. 1*B* and *C*). For the box H/ACA domain, helices P7a and P8b contain most conserved residues for maintaining the helical structure (fig. 1*B* and *C*). The CAB box located at the distal loop of P8 stem is conserved across most of the vertebrates (Reichow et al. 2007; Theimer et al. 2007) and echinoderm TRs (Podlevsky et al. 2016b) but absent in teleost fish TRs (Xie et al. 2008).

**Fig. 2.**
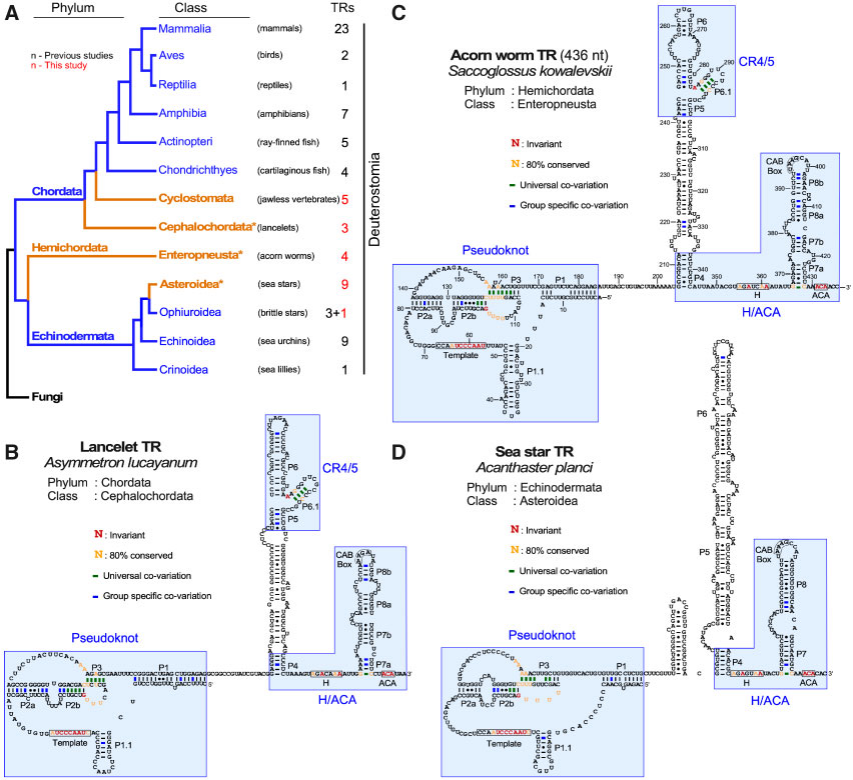
Secondary structures of deuterostome TRs. (*A*) Evolutionary relationship of deuterostome phyla and classes with TR identified. The numbers of TRs identified in this and previous studies for each class are indicated. An asterisk denotes the classes for which the TR secondary structure of a representative species is shown in *B*–*D*. Fungi represents the outgroup in the phylogenetic tree. (*B*–*D*) Representative TR secondary structures determined by phylogenetic comparative sequence analyses are shown for *Asymmetron lucayanum* (lancelet) from phylum chordata (*B*), *Saccoglossus kowalevskii* (acorn worm) from phylum hemichordata (*C*), and *Acanthaster planci* (sea star) from phylum Echinodermata (*D*). The conserved TR structural domains are shaded in blue. Universal covariations (thick lines), invariant residues, and residues with >80% conservation are based on the sequence alignment of 55 previously identified animal TRs and 82 novel metazoan TRs identified in this study. Group-specific covariations (thick lines) are indicated and based on the sequence alignment of TRs from individual groups including 50 chordate TRs (42 previously identified and eight novel), 23 echinoderm (13 previously identified and ten novel), and four acorn worm TRs.

Our initial TR homolog search targeted the unexplored species in deuterostomes, specifically the basal chordate groups. We trained the INFERNAL program with a PWM pattern generated from the sequence alignment of the conserved pseudoknot and box H/ACA regions together with the well-defined secondary structural constraints (fig. 1*C*). This initial search successfully identified TR candidates from the genome or transcriptome sequence data of five Cyclostomata (jawless fish) and three Cephalochordata (primitive fish-like eels) species (fig. 2*A*). The sequences of these eight putative TRs were aligned with the vertebrate chordate and echinoderm TR sequences to improve the diversity of the INFERNAL inquiry sequence and secondary structure pattern (fig. 1*A*). The bioinformatics searches using this improved INFERNAL search pattern, but not the initial pattern, successfully identified new TR sequences from 14 additional deuterostome species, including four acorn worm species from the hemichordate phylum and nine new echinoderm species from the starfish class asteroidea and one from the brittle star class ophiuroidea (fig. 2*A*).

### Deuterostome TRs Demonstrate the Evolutionary Transition of Telomerase TBE

We then deduced secondary structures of the newly identified deuterostome TRs through sequence alignment with vertebrate TRs that have their secondary structures well established (fig. 2*B*–*D*). The pseudoknot and box H/ACA structural domains of vertebrate TRs are universally conserved throughout deuterostomes (supplementary fig. S2*A*–*C*, Supplementary Material online). Although the vertebrate CR4/5 domain is conserved in basal chordate and hemichordate TRs (fig. 2*B* and *C*), the starfish TRs, similar to other echinoderm TRs, comprises an eCR4/5 domain, instead of the canonical CR4/5 domain (fig. 2*D*). In addition to a diversified eCR4/5, echinoderm TRs also possess a template-adjacent stem P1.1 that functions as a TBE and absent in a vast majority of vertebrate TRs (Podlevsky et al. 2016b). Comparative analysis of all deuterostome TR secondary structures showed that this P1.1-type TBE is ancestral, present in all echinoderm, hemichordate and early-branching chordate TRs but largely absent in vertebrate TRs, suggesting an evolutionary transition from the P1.1-type mechanism to the vertebrate P1 stem for defining the template boundary (figs. 1*B* and 2*B*–*D*).

### Functional Validation of the Deuterostome Acorn Worm TR

**Fig. 3.**
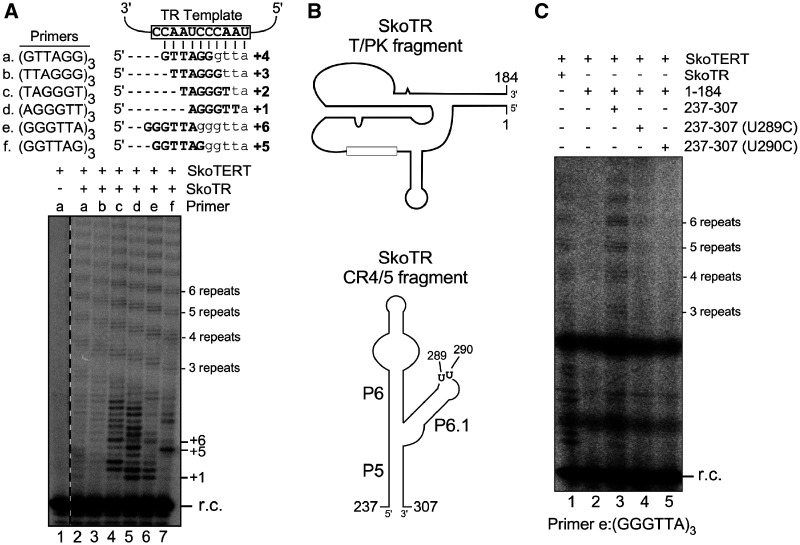
Functional validation and characterization of acorn worm TR. (*A*) Direct telomerase activity assay of acorn worm telomerase reconstituted in vitro. (top) Template sequence of acorn worm TR (open box) with base-pairing of six permuted telomeric DNA primers a–f. Sequence and number of expected nucleotides added are depicted for each primer. (bottom) Direct primer-extension assay of acorn worm telomerase. Acorn worm telomerase was in vitro reconstituted from T7 transcribed SkoTR (436 nt) and SkoTERT synthesized in RRL. The reconstituted acorn worm telomerase was analyzed with six permuted telomeric DNA primers (lanes 2–7). A reaction omitting SkoTR was included as a negative control (lane 1). A ^32^P end-labeled 18-mer oligonucleotide was added to each reaction as recovery control (r.c.) prior to ethanol precipitation of DNA products. Numbers to the right of the gel indicate the number of repeats or nucleotides added to the primer. (*B*) Two essential fragments of SkoTR. The T-PK and CR4/5 fragments of SkoTR were synthesized separately and assembled with SkoTERT in RRL, followed by telomerase activity assay. The schematic secondary structures of the SkoTR T-PK (top) and CR4/5 (bottom) fragments. Nucleotide numbers denote the 5′- and 3′-ends of the T7 transcribed RNA fragments, T-PK and CR4/5. Positions of two highly conserved U residues (U289 and U290) in the P6.1 loop of CR4/5 domain are shown. (*C*) Minimal requirement of TR domains for telomerase activity. T7 transcribed SkoTR fragments, T-PK (nt 1–184) and CR4/5 (nt 237–307), were assembled with in vitro synthesized SkoTERT and analyzed for activity. The CR4/5 fragments with a P6.1 substitution (U289C or U290C) were assembled with T-PK fragment and SkoTERT and assayed for activity with the primer (GGGTTA)_3_. A ^32^P end-labeled 18-mer oligonucleotide was added to each reaction prior to ethanol precipitation of DNA products as r.c. The numbers of repeats added to the primer are shown to the right of the gel.

To validate the newly identified deuterostome TRs, we cloned and synthesized the *TR* gene from *Saccoglossus kowalevskii* (acorn worm) as a representative from phylum hemichordata, distinct from the previously studied chordata and echinodermata phyla (fig. 2*A*). The *S. kowalevskii* TR (SkoTR) is 436 nt in length with the 5′-end determined by rapid amplification of cDNA ends (RACE) and the 3′-end assigned at three nucleotides downstream of the ACA box (fig. 2*C*). In addition to SkoTR, we also cloned the *S. kowalevskii* *TERT* (*SkoTERT*) gene. The in vitro synthesized SkoTERT and in vitro transcribed SkoTR were assembled in rabbit reticulocyte lysate (RRL) and analyzed for telomerase activity by direct primer-extension assay (see Materials and Methods). The telomerase activity assay generated telomeric DNA products with a characteristic ladder pattern with 6-nt increments (fig. 3*A*). This activity is SkoTR-dependent as no activity was detected in the absence of SkoTR (fig. 3*A*, lane 1). When assayed with six different DNA primers with permuted telomeric sequences, the activity generated DNA products with ladder patterns offset by a single nucleotide, indicating that a defined template was used for DNA synthesis (fig 3*A*, lanes 2–7). Collectively, these results functionally validated the SkoTR as the authentic RNA component of acorn worm telomerase.

To determine if the hemichordate CR4/5 is functionally homologous to vertebrate CR4/5, we assayed the acorn worm telomerase reconstituted with two separate SkoTR fragments, T-PK and CR4/5 (fig. 3*B*). Similar to the vertebrate CR4/5 that binds TERT independently (Tesmer et al. 1999; Mitchell and Collins 2000; Chen et al. 2002), the SkoTR CR4/5 fragment reconstituted telomerase activity in trans with the SkoTR T-PK fragment (fig. 3*C*, lanes 2 and 3). Moreover, the highly conserved U residues in the P6.1 loop of the human TR CR4/5 are crucial to telomerase activity (Chen et al. 2002). Similar point mutations, U289C and U290C (fig. 3*B*), introduced to the P6.1 loop of the SkoTR CR4/5 domain also abolished telomerase activity (fig. 3*C*, lanes 4 and 5), supporting that the hemichordate CR4/5 is functionally homologous to the vertebrate CR4/5 domain.

### Identification and Functional Validation of TR Homologs in Protostomes

**Fig. 4.**
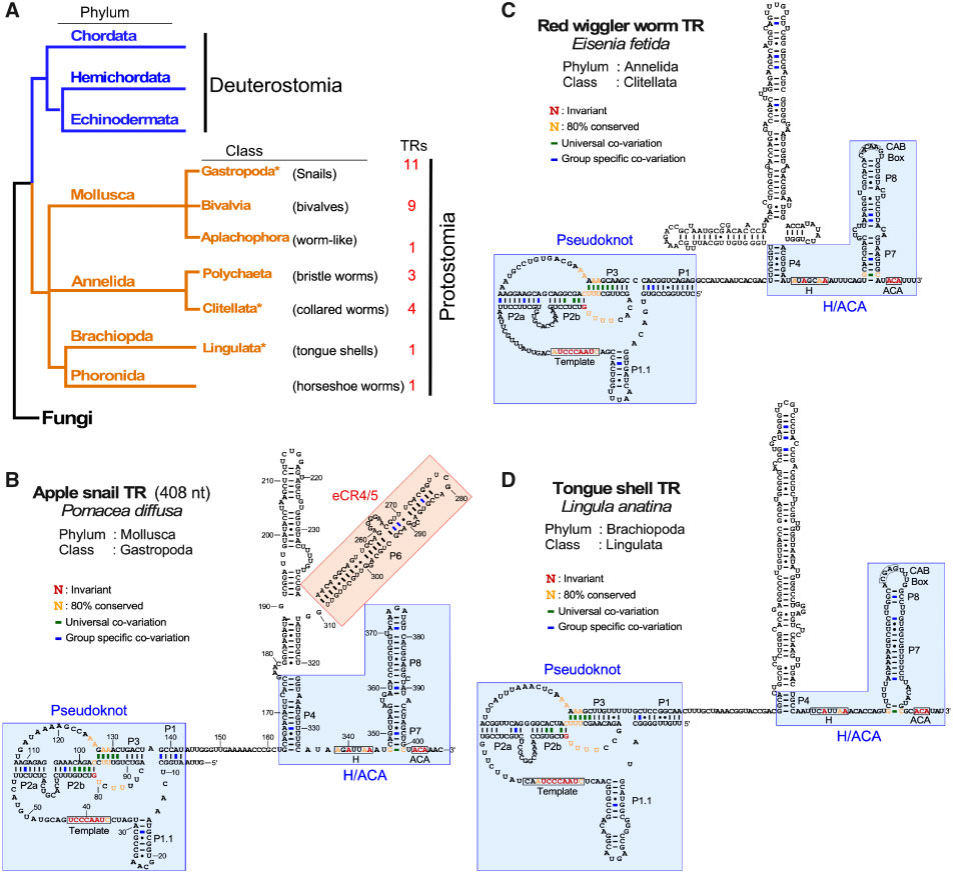
Secondary structures of protostome TRs. (*A*) Evolutionary relationship of protostome phyla and classes with TR identified. The numbers of TRs identified in this study for each class are indicated. An asterisk denotes the classes for which the TR secondary structure of a representative species is shown in *B*–*D*. Fungi represents the outgroup in the phylogenetic tree. (*B*–*D*) Representative TR secondary structures determined by phylogenetic comparative sequence analyses are shown; *Pomacea diffusa* (apple snail) from phylum mollusca (*B*), *Eisenia fetida* (earth worm) from phylum annelida (*C*), and *Lingula anatina* (tongue shell) from phylum brachipoda (*D*). The conserved TR structural domains are shaded in blue. The eCR4/5 domain is shaded in red. Universal covariations (thick lines), invariant residues, and residues with >80% conservation are based on the sequence alignment of 55 previously identified animal TRs and 82 novel metazoan TRs identified in this study. Group-specific covariations (thick lines) are indicated and based on the sequence alignment of TRs from individual groups including 21 mollusca, 7 annelida, 1 brachiopda, and 1 phoronida TRs, respectively.

Upon exhausting TR searches in available deuterostome sequencing data, we proceeded to search TRs in protostomes—the sister group to deuterostomes within bilateria (fig. 4*A*). During the course of TR homolog search, we continuously updated the multiple-sequence alignment with each newly identified TR sequence to improve the PWM patterns (fig. 1*A*). Using progressively improved PWM patterns for INFERNAL searches, we identified a total of 30 putative protostome TR sequences from four major phyla: 21 from mollusca, seven from annelida, one from brachiopoda, and one from phoronida (fig. 4*A*). In addition to these four phyla, we have also attempted TR searches in three distantly related protostome phyla, platyhelminthes (flatworms), arthropoda, and nematoda, which however failed to produce any convincing TR candidates.

Similar to the deuterostome TRs, the protostome TRs comprised a conserved T-PK core for providing the template and a box H/ACA domain for biogenesis (fig. 4*B*–*D* and supplementary fig. S3*A*–*C*, Supplementary Material online). In the T-PK core domain, the protostome TRs harbor a template proximal P1.1 stem similar to those in the nonvertebrate deuterostome TRs. However, the protostome TRs lack the vertebrate CR4/5 domain (fig. 4*B*–*D*). Instead, they possess one or two long stems connected to the P4 stem between the T-PK and box H/ACA domains with no sequence similarity to the canonical CR4/5 domain (fig. 4*B*–*D*).

**Fig. 5.**
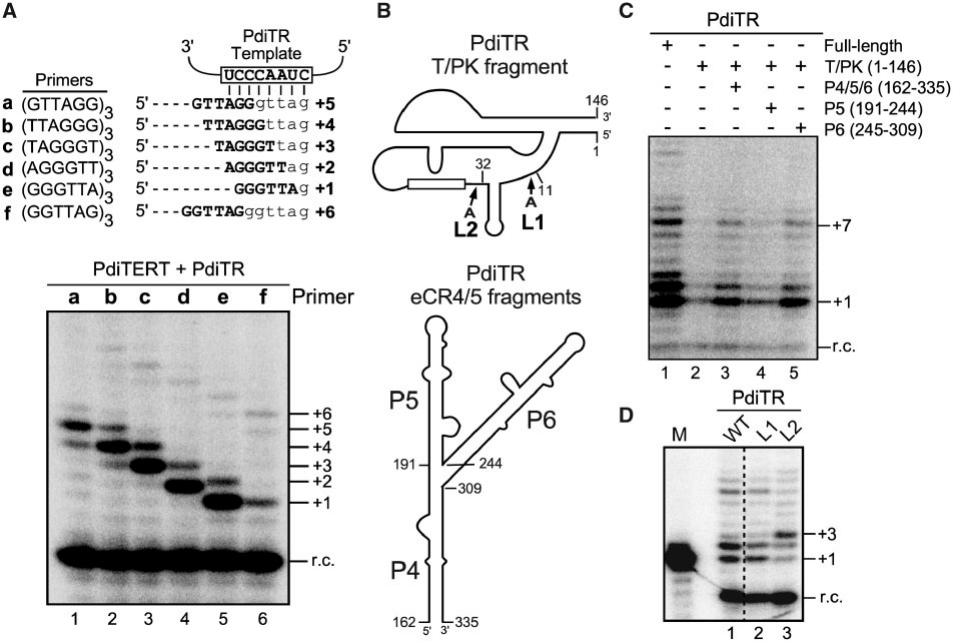
Functional validation and characterization of apple snail TR. (*A*) Direct telomerase activity assay of acorn worm telomerase reconstituted in vitro. (top) Template sequence of acorn worm TR (open box) with base-pairing of six permuted telomeric DNA primers a–f. Sequence and number of expected nucleotides added are depicted for each primer. (bottom) Direct primer-extension assay of apple snail telomerase. Apple snail telomerase was in vitro reconstituted from T7 transcribed PdiTR (408 nt) and PdiTERT synthesized in RRL. The reconstituted acorn worm telomerase was analyzed with six permuted telomeric DNA primers (lanes 1–6). A ^32^P end-labeled 18-mer oligonucleotide was added to each reaction as recovery control (r.c.) prior to ethanol precipitation of DNA products. Numbers to the right of the gel indicate the number of repeats or nucleotides added to the primer. (*B*) Two essential fragments of PdiTR. The T-PK and CR4/5 fragments of PdiTR were synthesized separately and assembled with PdiTERT in RRL, followed by telomerase activity assay. The schematic secondary structures of the PdiTR T-PK (top) and eCR4/5 (bottom) fragments. The eCR4/5 domain consists of three stems, P4–P6. Nucleotide numbers denote the 5′- and 3′-ends of the T7 transcribed TR fragments, T-PK, P4/5/6, P5, and P6. (*C*) Minimal requirement of TR domains for telomerase activity. T7 transcribed PdiTR fragments, T-PK (nt 1–146), P4/5/6 (nt 162–335), P5 (nt 191–244), or P6 (nt 245–309), were assembled with in vitro synthesized PdiTERT and analyzed for activity using the primer (GGGTTA)_3_. The PdiTR fragments included in each reaction are indicated above the gel. A ^32^P end-labeled 18-mer oligonucleotide was added to each reaction prior to ethanol precipitation of DNA products as r.c. The number of nucleotides added to the primer is shown to the right of the gel. (*D*) The effect of P1.1 position on template boundary definition. Two PdiTR mutants, L1 and L2, with a single adenosine residue inserted immediately after positions 11 and 32, respectively, were assembled in vitro with PdiTERT and assayed for telomerase activity using primer (GGGTTA)_3_.

To validate the newly identified protostome TRs, we cloned the *TR* gene from a mollusk, *Pomacea diffusa* (apple snail), as a representative from class gastropoda for detailed functional characterization (fig. 4*A*). The 5′- and 3′-RACE analyses determined the size of *P. diffusa* TR (PdiTR) to be 408 nt (fig. 4*B*). We also identified and cloned the *P. diffusa* *TERT* (*PdiTERT*) gene for in vitro synthesis using RRL. Apple snail telomerase was reconstituted in vitro by assembling synthesized PdiTERT and T7 transcribed PdiTR in RRL and analyzed for telomerase activity using direct primer-extension assay (see Materials and Methods). The reconstituted apple snail telomerase incorporated radioactive ^32^P-deoxyguanosine triphosphate (dGTP) and elongated the six permuted telomeric DNA primers, a–f, with expected numbers of nucleotide residues using the 8-nt template sequence, which produced the expected 1-nt offset in the banding patterns of the extended DNA products (fig. 5*A*). This template-directed primer-extension activity validates PdiTR as an authentic TR component from a protostome species (fig. 5*A*). Furthermore, the apple snail telomerase reconstituted in vitro had a significantly low processivity for repeat addition, as indicated by the low intensity of the second telomeric repeat synthesized, for example, the +7-nt band with primer e (fig. 5*A*, lane 5). It has been previously reported with human and mouse telomerases that the repeat addition processivity of telomerase positively correlates to the TR template length (Chen and Greider 2003a). Thus, the low processivity of apple snail telomerase is likely due to the short 8-nt template in PdiTR, which is significantly shorter than the 11-nt template in human TR (fig. 5*A*).

### Protostome Apple Snail TR Contains a Functional eCR4/5 Domain

The apple snail TR lacks the vertebrate CR4/5 domain and, instead, possesses two long stems, P5 and P6, connected to the P4 stem (fig. 4*B*). In order to discern if P5 or P6 stem is a functional replacement of the vertebrate CR4/5 domain for stimulating telomerase activity, we reconstituted apple snail telomerase using two separate PdiTR fragments, the T-PK (nt 1–146) and a second RNA fragment that includes either all three stems P4/5/6 (nt 162–335), P5 stem (nt 191–244), or P6 stem only (nt 245–309) (fig. 5*B*). The PdiTR fragments were assembled in trans with the synthetic PdiTERT protein in RRL and analyzed for telomerase activity using direct primer-extension assay (see Materials and Methods). Although the PdiTR T-PK fragment alone produced a basal level of activity with PdiTERT (fig. 5*C*, lane 2), the addition of the P4/5/6 or P6 fragment significantly stimulated telomerase activity (fig. 5*C*, lanes 3 and 5). In contrast, the addition of the P5 fragment did not show any stimulatory activity, indicating that the P6 stem in apple snail TR is a functional equivalent of the vertebrate CR4/5 element, that is, an eCR4/5 (fig. 5*C*, lane 4). However, it is noted that the T-PK and P4/5/6 fragments together did not reconstitute the full level of activity compared with the full-length PdiTR (fig. 5*C*, compare lanes 1 and 3), possibly due to a suboptimal condition for TR fragment folding or telomerase RNP assembly.

In addition to the eCR4/5 domain, we tested the function of stem P1.1 as a TBE. In echinoderm TR, the P1.1 stem defines the template boundary (Podlevsky et al. 2016b). We examined two PdiTR mutants, L1 and L2, that have a single adenosine residue inserted immediately after positions 11 and 32, respectively (fig. 5*B*). The banding pattern of primer-extension activity with telomerase reconstituted from PdiTR-L2 mutant showed nucleotide incorporation beyond the template boundary (fig. 5*D*, lane 3). The L2 mutant contains an insertion between the template and stem P1.1, which shifted the position of stem P1.1. Thus, stem P1.1 in apple snail TR functions as a TBE and is conserved throughout protostomes and nonvertebrate deuterostomes.

### The Basal Metazoan TR Preserves the Ancestral Core Structural Domains

**Fig. 6.**
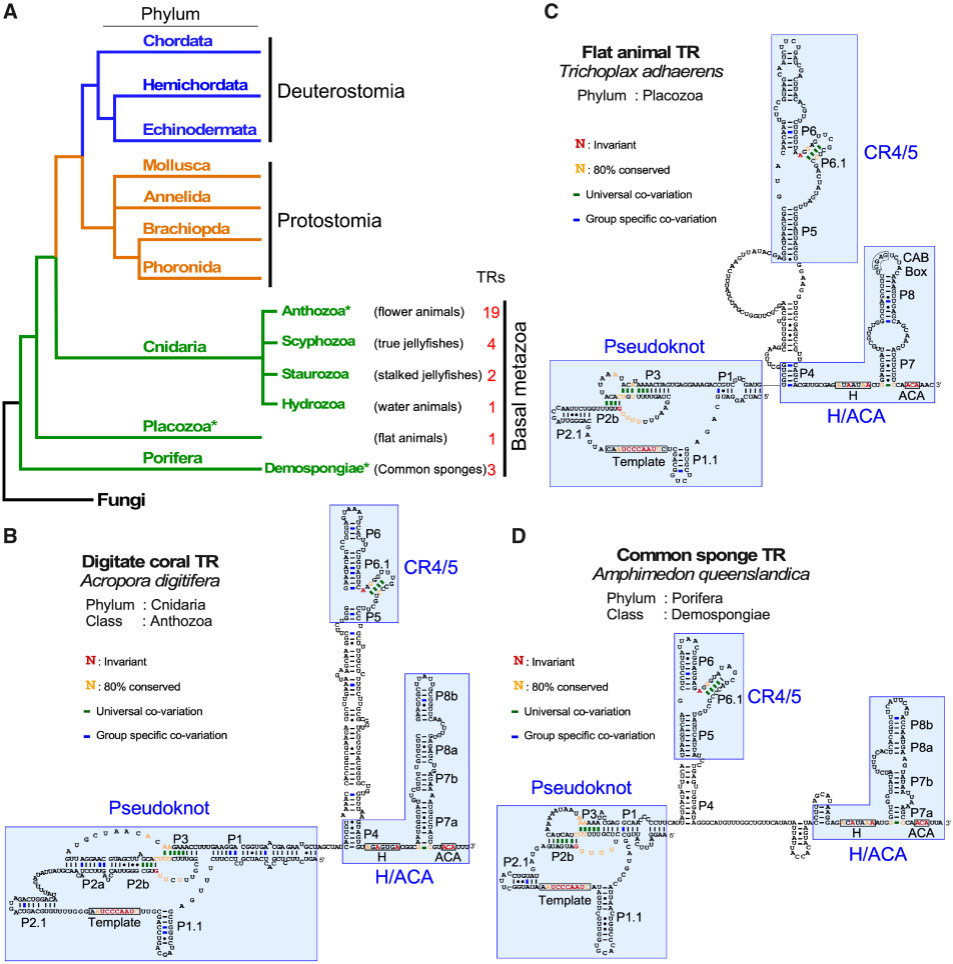
Secondary structures of basal metazoan TRs. (*A*) Evolutionary relationship of basal metazoan phyla and classes with TR identified. The numbers of TRs identified in this study for each class are indicated. An asterisk denotes the classes for which the TR secondary structure of a representative species is shown in *B*–*D*. Fungi represents the outgroup in the phylogenetic tree. (*B*–*D*) Representative TR secondary structures determined by phylogenetic comparative sequence analyses are shown for *Acropora digitifera* (digitate coral) from phylum cnidaria (*B*), *Trichoplax adhaerens* (flat animal) from phylum placozoa (*C*), and *Amphimedon queenslandica* (common sponge) from phylum porifera (*D*). The conserved TR structural domains are shaded. Universal covariations (thick lines), invariant residues, and residues with >80% conservation are based on the sequence alignment of 55 previously identified animal TRs and 82 novel metazoan TRs identified in this study. Group-specific covariations are indicated and based on the sequence alignment of TRs from individual groups including 26 cnidaria, 1 placozoa, and 3 porifera species.

In addition to protostomes and deuterostomes, we expanded our TR search into the most basal metazoan groups including three major phyla: cnidaria (nettles), placozoa (flat animals), and porifera (sponges) (fig. 6). In the cnidarian phylum, we identified putative TR sequences from 26 species that belong to four classes: 19 anthozoa (sea anemones and corals), four scyphozoa (true jellyfishes), two staurozoa (stalked jellyfishes), and one hydrozoa (water animals). Furthermore, we identified one TR in the placozoa phylum of flat animals and three TRs in the porifera phylum of sponges, the most basal living metazoan organisms (fig. 6*A*).

For these basal metazoan groups, we inferred TR secondary structure of a representative species from each of the cnidaria, placozoa, and porifera phyla (fig. 6*B*–*D*). The basal metazoan TR preserved the T-PK, CR4/5, and box H/ACA core domains that are ubiquitously conserved in other major metazoan phyla. In addition, the universal presence of stem P1.1 in basal metazoan TRs suggested that the P1.1-type TBE is ancestral (fig. 6*B*–*D* and supplementary fig. S5*A*–*C*, Supplementary Material online). Moreover, the T-PK domains of basal metazoan TRs contain an additional stem termed P2.1. Most importantly, all basal metazoan TRs contain the canonical CR4/5 domain with the highly conserved P6.1 stem-loop which is present throughout most major metazoan lineages, except the echinoderms and protostomes (figs. 1*B* and 4*B*–*D*). The conservation of a canonical CR4/5 domain in basal metazoan TRs indicates that the CR4/5 is an ancestral structural element but was replaced by eCR4/5 in echinoderms and protostomes. Towards experimentally validating representative basal metazoan TRs, we cloned both *TERT* and *TR* genes from two sponges, *Amphimedon queenslandica* and *Oscarella carmela*, to attempt in vitro telomerase enzyme reconstitution (see Materials and Methods). Although we were able to express *A. queenslandica* and *O. carmela* TERT proteins in RRL (supplementary fig. S6*A* and *B*, Supplementary Material online), we did not detect telomerase activity from the in vitro reconstituted enzyme.

## Discussion

Telomerase emerged in early eukaryotes as a highly specialized reverse transcriptase with an integral RNA providing the template for telomeric DNA repeat synthesis. Over the past three decades, the TR component has been extensively studied in a few groups of eukaryotes including vertebrates, echinoderms, fungi, plants, and ciliates, demonstrating unusual divergence in structure and biogenesis pathway among eukaryotic kingdoms (Podlevsky and Chen 2016). Within the animal kingdom, only vertebrates and echinoderms have had their TRs identified and studied (Chen et al. 2000; Xie et al. 2008; Li et al. 2013; Podlevsky et al. 2016b). The lack of TR identified in the vast majority of metazoan phyla drastically hinders a kingdom-wide investigation of TR evolution. In this study, we overcome the challenge of TR sequence divergence by employing an effective phylogeny-assisted structure-homology search strategy and successfully identified 82 novel animal TRs from a broad range of disparate metazoan clades including the most basal sponge species.

Our fruitful TR discovery approach leverages the sequence and structural homology inferred from known TRs and targets initially the phylogenetically close-related species. It is worth noting that conventional sequence-homology search tools such as BLAST (Altschul et al. 1990) are limited to TR identification from a handful number of species that are phylogenetically closely related. A more advanced sequence-homology search method such as fragrep 2 (Mosig et al. 2007) that uses PWMs derived from multiple-sequence alignments, achieves limited success in finding TR from some phylogenetically closely related species (Xie et al. 2008; Podlevsky et al. 2016b), but not in those distantly related species. Our bioinformatics strategy searches for both sequence and structural conservation of TRs using INFERNAL (Nawrocki and Eddy 2013). Initially, multiple-sequence alignment of TRs with structural annotations is generated using existing secondary structure information either supported experimentally and/or via covariation. A statistical model of the alignment which considers both secondary structure information and position-specific sequence conservation known as a covariance model is generated using INFERNAL. This model is then used to search against the genome or transcriptome of a phylogenetically closely related target species to obtain TR candidates. Secondary structure model and primary sequence alignment is used to verify the hits to identify a bona fide TR. This process is reiterated after generating an improved covariance model by including the newly identified TR and searching for TRs from organisms in the next closely related clade (fig. 1*A*). Careful secondary structural analysis and strategic search based on well-established phylogenetic relationships are keys in finding TR sequences in previously unexplored group of species, which would otherwise prove extremely tedious with conventional methods.

Telomerases from all known eukaryotes show a functional requirement of two TR structure domains, T-PK and CR4/5 (or eCR4/5-a functional equivalent of CR4/5), for enzymatic activity (Podlevsky and Chen 2016). As demonstrated in vertebrate, fungal, plant, ciliate, and even flagellate trypanosome telomerases, these two TR domains can bind independently to the TERT protein to reconstitute activity in vitro (Tesmer et al. 1999; Mitchell and Collins 2000; Mason et al. 2003; Qi et al. 2013; Podlevsky et al. 2016a, 2016b; Song et al. 2019). In vertebrates, the CR4/5 domain has high binding affinity toward the TERT-TRBD which relies on specific interacting residues between the two components (Bley et al. 2011; Huang et al. 2014). For the T-PK domain, although the recent cryo-EM structures of tetrahymena and human telomerase complexes have provided crucial insight into the T-PK binding surface on TERT (Jiang et al. 2015, 2018; Nguyen et al. 2018), the interacting residues mediating the T-PK and TERT binding remain elusive due to the lack of high-resolution details. Interestingly, the dependence for in vitro activity on these TR domains is variable among different kingdoms. For instance, vertebrate and filamentous fungal telomerases require both T-PK and CR4/5 domains to be present for full activity. However, for flagellate and echinoderm telomerases, the T-PK domain alone reconstitutes ∼30–40% of full activity when assembled with the TERT protein, and the distal stem-loop eCR4/5 moiety is required for reconstituting full activity, suggesting a lower functional dependence of the eCR4/5 domain (Podlevsky et al. 2016a, 2016b). However, ciliate TRs show only partial activity if two TR fragments are added in trans compared to full-length TR (Mason et al. 2003). This is potentially due to the compact nature of ciliate TRs which promotes a functional codependence between the two TR domains.

In this study, we show that acorn worm and apple snail telomerases also require two separate TR domains to reconstitute telomerase activity in vitro (figs. 3*B*, 3*C*, 5*B*, and 5*C*), suggesting that this two TR domain requirement is a universal attribute of telomerase function across eukaryotes. Although acorn worm TR contains the canonical CR4/5 domain, the apple snail TR contains an eCR4/5 domain similar to the echinoderms. Telomerase from the basal metazoan groups ubiquitously contains a canonical CR4/5 domain and is likely to preserve the two-domain requirement for in vitro telomerase activity. As a means to demonstrate this, we cloned the *TERT* and *TR* genes from two sponge species, *A. queenslandica* and *O. carmela* and successfully expressed the sponge TERTs in the RRL. However, our attempts to demonstrate in vitro telomerase activity was unsuccessful potentially due to lack of conservation in RNP assembly chaperones between sponges and vertebrates. Although the origin of specific domains in the TR is unclear, we speculate that the two-domain requirement would prevent the ancient TERT protein from promiscuous and detrimental DNA synthesis using nonspecific RNA molecules as template. Thus, through acquiring the two essential TR domains, telomerase emerged in early eukaryotes as an RNP enzyme distinct from the conventional RT protein enzymes.

**Fig. 7.**
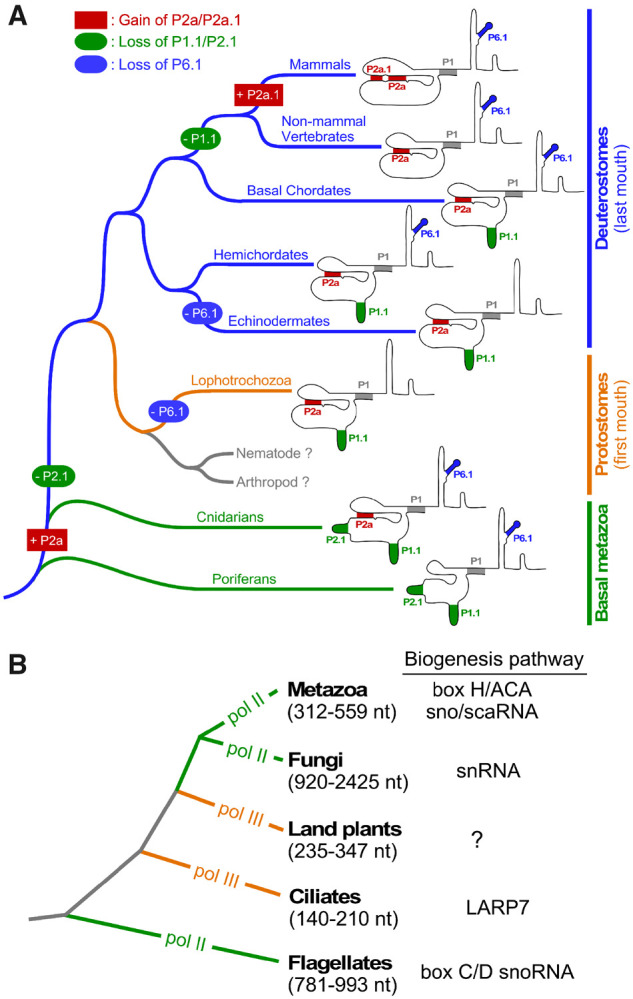
Monophyletic origin and divergent evolution of TR structural elements and biogenesis pathways. Simplified phylogenetic trees of major metazoan (*A*) and eukaryotic (*B*) lineages shown. Branch lengths do not represent evolutionary distance. (*A*) Evolution of TR structural elements across metazoan lineages. Loss of RNA secondary structural elements P1.1, P2.1, or P6.1 is indicated in oval. Gain of P2a or P2a.1 is shown in rectangle. Specific events of loss and gain of TR structural elements are marked along the respective metazoan lineages. (*B*) Divergence of TR biogenesis pathway and transcription machinery across eukaryotes. The TR transcription machinery in each eukaryotic kingdom is indicated as Pol II or Pol III. The size range of TRs from each group is indicated. Biogenesis pathway of respective eukaryotic clades are shown to the right.

The conservation of all three core domains, T-PK, CR4/5, and H/ACA, in both vertebrate and sponge TRs indicates a monophyletic origin of animal telomerase (fig. 7*A*). Although the H/ACA domain remains unchanged, the T-PK and CR4/5 domains exhibit gradual gain or loss of helical elements during evolution along distinct metazoan lineages. For example, the T-PK domain comprises a number of structural elements, in addition to the template, that play specific roles in controlling telomerase function. The template-adjacent helix P1.1 is a ubiquitous TBE that emerged early in metazoan lineages such as cnidarians and poriferan sponges and remained conserved in all protostomes and most deuterostomes including basal chordates (fig. 7*A*). This ancestral P1.1 stem is also conserved in fungal, plant, and ciliate TRs as TBE, preventing template read-through and addition of nontelomeric DNA sequences (Tzfati et al. 2000; Lai et al. 2002; Qi et al. 2013; Song et al. 2019). The template proximal helix type TBE mostly prevents template bypass by limiting the availability of single-stranded RNA for DNA repeat synthesis, while ciliate TRs contain conserved residues at the base of helix II stem that bind TERT and prevent usage of nontemplate sequences (Jansson et al. 2015). The ancestral P1.1-type TBE was then replaced with a P1-mediated mechanism in vertebrates (Chen and Greider 2003b), which relies on the linker between the template and the P1 stem to control the movement of the template (fig. 7*A*). Surprisingly, hagfish and lampreys that are vertebrates, seem to use the P1.1-type TBE (supplementary fig. S2*C*, Supplementary Material online). This suggests the switch from P1.1- to P1-type TBE is a recent event specific to more later evolving vertebrates in TR structural evolution (supplementary fig. S2*C*, Supplementary Material online). Moreover, it has been previously demonstrated that the P1.1 helix in echinoderms can be deleted completely and the telomerase switches to the P1 type template boundary and vice versa (Podlevsky et al. 2016b). This suggests that the switch between P1 and P1.1 type is rather plastic. However, based on secondary structural models of protostomes and basal metazoan TRs, the P1.1 type is universal suggesting P1.1 is favored over P1 type. For instance, as species-specific insertions between the template and P1 occur, local structures such as P1.1 are formed to limit the linker length between the template and P1. The extension of stem P2 with stem P2a in cnidarian and stem P2a.1 in mammals or loss of stem P2.1 in protostomes in the T-PK domain demonstrates structural evolution of TR throughout metazoan evolution (fig. 7*A*).

Compared with the T-PK domain, the CR4/5 domain shows higher diversity in its function and structure. The vertebrate-type CR4/5 structure with the highly conserved P6.1 stem-loop can be found in the basal-branching sponges and filamentous fungi (Qi et al. 2013), but not plants (Song et al. 2019), suggesting that the CR4/5 domain is ancestral predating the split between the fungal and metazoan lineages (fig. 6*A*). However, CR4/5 appears to diversify to an eCR4/5 element with the loss of P6.1 stem twice in the metazoan kingdom, once along the protostomia lineage and the second time along the echinoderm lineage (fig. 7*A*). Interestingly, TRs from hemichordates, a sister clade of echinoderms, preserve the canonical CR4/5 domain (fig. 7*A*). The diversification of CR4/5 in distinct metazoan lineages presumably requires coevolution with the TRBD domain of TERT to accommodate the simpler eCR4/5 structures. We have previously identified the interacting residues between medaka fish TRBD and CR4/5 using an approach combining UV cross-linking and mass spectrometry (Bley et al. 2011), as well as by determination of a medaka TRBD-CR4/5 co-crystal structure (Huang et al. 2014). To understand how TRBD and eCR4/5 co-evolved to maintain their functional interactions would require a high-resolution structure of a TRBD-eCR4/5 complex to reveal the interacting residues at the binding interface. Our study provides a plethora of potential systems for TRBD-eCR4/5 binding studies and ultimately RNP co-crystal structure determination.

Although the T-PK and CR4/5 domains are conserved for enzymatic function, the rest of the TR comprises of intriguingly divergent structural elements crucial for telomerase biogenesis and regulation in vivo among different eukaryotic kingdoms primarily as a result of the variable transcription machinery (fig. 7*B*). The TR biogenesis in distinct groups of species shares distinct sets of protein components with known noncoding RNA families including the box C/D snoRNA in trypanosomes (Gupta et al. 2013), small transcripts of RNA polymerase III in ciliates (Witkin and Collins 2004), snRNA in fungi (Seto et al. 1999), and box H/ACA sno/scaRNA in vertebrates (Mitchell et al. 1999; Jády et al. 2004; Venteicher et al. 2009; Li et al. 2013). The dramatic variation of TR size and structural domains provides scaffold for binding various classes of RNA-binding accessory proteins for distinct biogenesis pathways employed by different groups of eukaryotic species (Podlevsky and Chen 2016). Moreover, the transcription by RNA polymerase III (pol III) explains the smaller sizes of ciliate and plant TRs (fig. 7*B*). However, the evolutionary transitions between RNA Pol II and Pol III in different lineages remain an unsolved mystery (fig. 7*B*). In this study, the presence of box H/ACA domain in all identified metazoan TRs including sponges suggests that the box H/ACA snoRNA-type RNA biogenesis emerged early during animal evolution, likely through adaptation of the box H/ACA structural domain as binding sites for the existing protein components of box H/ACA snoRNP complexes (fig. 6*B*–*D*). However, the conservation of the CAB box in the majority of basal metazoan TRs suggests that Cajal body localization of TR during biogenesis emerged early in metazoan kingdom (supplementary fig. S7, Supplementary Material online). Given the distinct biogenesis mechanisms utilized by TRs from the three neighboring kingdoms, plants, fungi, and animals, the origin and evolution of these TR biogenesis mechanisms awaits further study (fig. 7*B*).

Although our phylogeny-guided, structure-based TR identification strategy has been successfully applied to a wide range of metazoan phyla, the TRs from the nematoda and arthropoda phyla remain enigmatic despite exhaustive searches (fig. 7*A*). Within the arthropoda, although certain clades of insects utilize retrotransposon-mediated mechanism for telomere maintenance (Casacuberta 2017), many insect taxa contain uniform telomeric repeats of the noncanonical TTAGG sequence, suggesting a telomerase-mediated mechanism (Vítková et al. 2005). However, the deviation of telomeric repeat sequence from the prevalent TTAGGG sequence would require a longer evolutionary distance that lead to further diversification in TR primary sequence and secondary structure as demonstrated between the filamentous fungal and yeast TRs (Qi et al. 2013). As our TR search methodology relies on conservation of secondary structure to a greater extent, a vastly divergent sequence and structure may explain our inability to identify TRs in nematoda and arthropoda. Nonetheless, the conservation and diversification of TR secondary structure across the animal kingdom revealed by this work will provide important foundation for future elucidation of TRs from nematodes and arthropods, which will ultimately shed light on the unusual divergent evolution of the telomerase RNP across eukaryotes.

## Materials and Methods

### Multiple-Sequence Alignment

Sequence alignment of vertebrate and echinoderm TRs was performed initially using the ClustalW algorithm within the BioEdit program (Hall 1999). The alignments were then refined manually using previously identified or highly conserved sequence motifs as anchor points. The alignment started with sequences from closely related species and then expanded to include sequences from more distantly related species.

### RNA Isolation

Fresh tissues from *Pomacea giganteus* (gonad), *P. ochraceus* (gonad), *P. diffusa* (intestine), *S. kowalevskii* (whole body), and *Saccoglossus bromophenolosus* (whole body) were homogenized in TRI-Reagent (Molecular Research Center, Inc.), followed by acid phenol/chloroform extraction and ethanol precipitation. Integrity of isolated RNA was monitored by denaturing gel electrophoresis (1% agarose/formaldehyde) or analyzed by 2100 Bioanalyzer (Agilent).

### Next-Generation RNA-Seq

Ten micrograms of total RNA from *P. giganteus* and *P. ochraceus* were resolved by denaturing polyacrylamide gel electrophoresis (4% polyacrylamide/8 M urea). RNA species with sizes of 300–750 nt were excised from the gel and eluted, followed by ethanol precipitation. Size-selected RNA was used for cDNA library construction with the ScriptSeq v2 RNA-seq Library Preparation Kit (Epicentre) following the manufacturer’s instructions. The cDNA libraries were amplified using ScriptSeq Index PCR Primers (Epicentre) and the indexed cDNA libraries were pooled and analyzed in a single multiplexed single-end 50-bp sequencing run on a HiSeq 2000 (Illumina).

### Bioinformatics Analysis

Searches for putative TR sequences from early-branching chordates and class asteroidea from echinoderms were performed using the INFERNAL (INFERence of RNA Alignments version 1.1.2, July 2016) program (Nawrocki and Eddy 2013; Barquist et al. 2016) with search patterns that consist of PWMs generated from the multiple-sequence alignment of 42 vertebrate and 13 echinoderm TRs (supplementary fig. S1, Supplementary Material online). To search TRs in distantly related species, the search pattern used for Infernal program was modified progressively based on updated multiple-sequence alignments that include newly identified TR sequences from closely related species. Publicly available data sources for the identification of TR sequences are listed in supplementary table S1, Supplementary Material online. For the identification of *P. giganteus* and *P. ochraceus* TRs, transcriptomes assembled from next-generation sequencing data of size-selected RNAs were used for Infernal search.

### Cloning of *TERT* and *TR* Genes

Partial *TERT* gene (GenBank accession no. of scaffold NW_003141316.1) of *S. kowalevskii* was identified from the *S. kowalevskii* genome (assembly Skow_1.1) by BLAST using *Strongylocentrotus purpuratus* (purple sea urchin) TERT as query (GenBank accession no. ACL80758.1). Partial TERT sequence of *P. diffusa was* obtained by BLAST search against *P. diffusa* protein database (Ampubase) using *Pomacea canaliculata* TERT-like isoform X1 (NCBI reference sequence no. XP_025094247.1) as query. The predicted TERT cDNA of *A. queenslandica* sponge (NCBI reference sequence no. XM_019994917.1) was synthetically generated. DNAWorks program (Hoover and Lubkowski 2002) was used to design 84 oligonucleotides (Integrated DNA Technologies) to span the open reading frame of the *A. queenslandica* TERT gene. The synthetic gene was constructed by “oligo shuffling” as previously described with minor modifications (Stemmer 1994). Briefly, oligonucleotides were pooled into four groups of 24 (4 µM each) and 1 µl was used in a 25 µl PCR reaction containing 1× Q5 reaction buffer (25 mM TAPS–HCl at pH 9.3, 50 mM KCl, 2 mM MgCl_2_, and 1 mM β-mercaptoethanol), 0.2 mM each 2'-deoxyribonucleoside triphosphate (dNTP), and 0.5 U of Q5 DNA Polymerase (NEB). One microliter of the previous PCR was then amplified in a second 25 µl PCR with 0.5 µM outermost oligonucleotides as primers. The *O. carmela* sponge TERT coding sequence (contig, comp39334_c0_seq80) was identified by tblastn search (standalone BLAST version 2.2.31+) using the *A. queenslandica* TERT open reading frame against a custom database generated using *O. carmela* transcript models (Hemmrich and Bosch 2008). The 5′- and 3′-ends of *S. kowalevskii* and *P. diffusa* TERTs and TRs were determined by RACE using the FirstChoice RLM-RACE kit (Ambion) following manufacturer’s instructions. For the 3′ RACE, total RNA was pretreated with poly(A) RNA polymerase (USB). For all other full-length metazoan TRs identified in this study, the 5′-end was predicted by the proximity of a TATA box for transcription initiation and a putative P1 helix with the 3′-end predicted 3 nt downstream from the box ACA motif as previously described (Chen et al. 2000; Podlevsky et al. 2016b). The experimentally determined *S. kowalevskii* and *P. diffusa* TR sequences were PCR amplified from genomic DNA. The *A. queenslandica* and *O. carmela* TR genes identified via INFERNAL search were created using synthetic oligonucleotides following the “oligo shuffling” method. All four TRs were cloned into the pCR4-TOPO vector and sequenced. The coding sequences of *S. kowalevskii* and *P. diffusa* TERTs were generated by RT-PCR. The *O. carmela* TERT was PCR amplified from cDNA library provided by Dr Scott Nichols (University of Denver, CO). *Saccoglossus kowalevskii*, *P. diffusa, A. queenslandica*, and *O. carmela* TERT coding sequences were subsequently cloned into the pCITE4a vector with an N-terminal 3XFLAG epitope tag and sequenced.

### Telomerase In Vitro Reconstitution

The FLAG-tagged acorn worm (SkoTERT) and apple snail (PdiTERT) TERTs were synthesized in RRL using the pFLAG-TERT plasmid and the TNT Quick Coupled transcription/translation kit (Promega) following manufacturer’s instructions. The RRL synthesis of SkoTERT was supplemented with 60–100 mM KCl depending on the batch of the RRL. The synthesis of PdiTERT in RRL was supplemented with 20 mM KCl. The TR fragments were in vitro transcribed by T7 RNA polymerase, gel purified, and assembled with TERT protein for 30 min at 30 °C at a final concentration of 1.0–1.5 µM.

### Telomerase Direct Primer-Extension Assay

Twelve microliters of in vitro reconstituted telomerase enzyme was immunopurified with 3 µl of anti-FLAG M2 magnetic beads (Sigma M8823) at room temperature for 1 h. The telomerase enzyme on beads was assayed in a 10-µl reaction containing 1× primer-extension (PE) buffer, 1 µM DNA primer, 100 µM deoxyadenosine triphosphate (dATP), 100 µM deoxythymidine triphosphate (dTTP), 5 µM cold deoxyguanosine triphosphate (dGTP), and 0.18 µM of ^32^P-dGTP (3,000 Ci/mmol, 10 mCi/ml, PerkinElmer). PE-D buffer (50 mM Tris–HCl, pH 8.0, 50 mM NaCl, 0.5 mM MgCl_2_, 5 mM β-mercaptoethanol, and 1 mM spermidine) was used for skoTERT, PE-18 buffer (50 mM Tris–HCl, pH 8.0, 4 mM MgCl_2_, 5 mM β-mercaptoethanol, and 1 mM spermidine) was used for PdiTERT. Reactions were incubated at 30 °C for 60 min and terminated by phenol/chloroform extraction, followed by ethanol precipitation and resuspended in 5 µl of 2× formamide loading buffer (10 mM Tris–HCl, pH 8.0, 80% [vol/vol] formamide, 2 mM ethylenediaminetetraacetic acid, 0.08% bromophenol blue, and 0.08% xylene cyanol). The 19-mer size marker was prepared by end-labeling a (GGGTTA)3 oligonucleotide in a 10-μl reaction containing five units of terminal deoxynucleotidyl transferase (TdT) (Affymetrix), 0.1 μM of ^32^P-dGTP and 1× TdT reaction buffer. The TdT reaction was incubated at room temperature for 3 s and terminated by addition of 10 μl 2× formamide loading buffer. The DNA products were resolved on a 10% (wt/vol) polyacrylamide/8 M urea denaturing gel, dried, and exposed to a phosphor storage screen and imaged on a Typhoon gel scanner (GE Healthcare).

## Supplementary Material

Supplementary data are available at *Molecular Biology and Evolution* online.

## Supplementary Material

msaa203_Supplementary_Data

## Notes

Illumina sequencing reads generated have been deposited at the NCBI Sequence Read Archive (SRA) database under the accession number PRJNA612126. All telomerase gene sequences have been deposited at the GenBank and Telomerase Database (https://telomerase.asu.edu; last accessed August 19, 2020).
