# Supplementary material for: Monophyletic Origin and Divergent Evolution of Animal Telomerase RNA
Source: Mol Biol Evol. 2020 Aug 8;38(1):215–28. doi: 10.1093/molbev/msaa203 (PMC8480181; doi:10.1093/molbev/msaa203)

**Supplementary figure legends**

**Supplementary Fig. S1.** Phylogenetic comparative analysis for position-specific weight matrix and secondary structure-based search for additional early branching chordate and echinoderm TRs. Multiple sequence alignment of 42 vertebrate (Chen et al. 2000) and 13 echinoderm (Li et al. 2013; Podlevsky et al. 2016b) TR pseudoknot (A) and box H/ACA (B) domains. Residues that are conserved 80% or more are shaded with white text on colored background. A dashed line separates vertebrates and echinoderms. Conserved primary sequence and secondary structural motifs are labeled below the alignment.

**Supplementary Fig. S2.** Phylogenetic comparative analysis based secondary structure determination of deuterostome TR domains. Multiple sequence alignment of 23 novel deuterostome TR pseudoknot domains (A) and box H/ACA domains (B). Residues conserved 80% or more are shaded with white text on colored background. Conserved primary sequence and secondary structural motifs are labeled below the alignment. (C) Secondary structure models of select novel deuterostome TR, pseudoknot and box H/ACA domains. Universal co-variations (green lines), invariant residues (red) and residues with >80% conservation (orange) are based on the sequence alignment of 55 previously identified animal TRs and 82 novel metazoan TRs identified in this study. Group-specific covariations (blue lines) are indicated and based on the sequence alignment of TRs from individual groups including 50 chordate TRs (42 previously identified and 8 novel), 23 echinoderm (13 previously identified and 10 novel) and 4 acorn worm TRs.

**Supplementary Fig. S3.** Phylogenetic comparative analysis based secondary structure determination of protostome TR domains. Multiple sequence alignment of 29 novel protostome TR pseudoknot (A) and box H/ACA domains (B). Residues conserved 80% or more are shaded with white text on colored background. Conserved primary sequence and secondary structural motifs are labeled below the alignment. (C) Secondary structure models of select novel protostome TR, pseudoknot and box H/ACA domains. Universal co-variations (green lines), invariant residues (red) and residues with >80% conservation(orange) are based on the sequence alignment of 55 previously identified animal TRs and 82 novel metazoan TRs identified in this study. Group-specific covariations (blue lines) are indicated and based on the sequence alignment of TRs from individual groups including 21 mollusca, 7 annelida, 1 brachiopda and 1 phoronida TRs, respectively.

**Supplementary Fig. S4.** Identification of metazoan TRs. A simplified phylogenetic tree representing major metazoan clades. Lineages are colored; Deuterostomia (blue),

Protostomia (orange) and basal metazoan (green). Branch lengths do not represent evolutionary distances. The number of previously identified TRs are shown in black to the right of respective classes and number of TRs identified in this study are shown in red. A total of 82 novel TRs were identified in this study spanning 10 metazoan phyla and 18 classes.

**Supplementary Fig. S5.** Phylogenetic comparative analysis based secondary structure determination of basal metazoan TR domains. Multiple sequence alignment of 29 novel basal metazoan TR pseudoknot (A) and box H/ACA domains (B). Residues conserved 80% or more are shaded with white text on colored background. Conserved primary sequence and secondary structural motifs are labeled below the alignment. (C) Secondary structure models of select novel basal metazoan TR, pseudoknot and box H/ACA domains. Universal co-variations (green lines), invariant residues (red) and residues with >80% conservation (orange) are based on the sequence alignment of 55 previously identified animal TRs and 82 novel metazoan TRs identified in this study. Group-specific covariations (blue lines) are indicated and based on the sequence alignment of TRs from individual groups including 26 cnidaria, 1 placozoa and 3 porifera species.

**Supplementary Fig. S6.** *In vitro* synthesis of sponge TERT proteins. The sponge TERT proteins were synthesized in RRL in the presence of $^{35}$S methionine and analyzed on SDS-PAGE gel, exposed to phosphorstorage screen, and imaged with a Typhoon gel imager (GE healthcare). (A) FLAG tagged *Amphimedon queenslandica* TERT, FLAG-AquTERT (lane 2) and FLAG tagged human TERT, FLAG-hTERT (lane 1) expressed as control and a size marker. (B) FLAG tagged *Oscarella carmela* TERT (OcaTERT).

**Supplementary Fig. S7.** Conservation of CAB box in metazoan TRs. Multiple sequence alignments of the distal P8 stem-loop of TRs from select representative species of each clade are shown. RNA secondary structure is indicated using dot-bracket notations above each alignment and the RNA structural element is labeled above base-pairing notations. Residues conserved 80% or more are shaded with white text on colored background. CAB box conservation is shown within red box and labeled above the box in red.

**Table S1. Species with TR identified in this study**

| Clade | Phylum | Class | Species | Accession | Start Coordinates[a] | End Coordinates[b] | Strand | Source |
|---|---|---|---|---|---|---|---|---|
| Deuterostomia | Chordata | Cyclostomata | *Petromyzon marinus* | PIZI01000001.1 | 7,205,787 | 7,206,194 | + | NCBI |
| | | Cyclostomata | *Lethenteron camtschaticum* | APJL01104664.1 | 144 | 547 | - | NCBI |
| | | Cyclostomata | *Lampetra planeri* | SRR5230929 | n/a | n/a | n/a | NCBI-SRA |
| | | Cyclostomata | *Eptatretus burgeri* | FYBX02010827.1 | 605,218 | 605,647 | - | NCBI |
| | | Cyclostomata | *Eptatretus atami* | DRR062483 | n/a | n/a | n/a | NCBI-SRA |
| | | Cephalochordata | *Asymmeteron lucayanum* | LZCU01053182.1 | 582 | 974 | + | NCBI |
| | | Cephalochordata | *Branchiostoma belcheri* | AYSS01005866.1 | 20,970 | 21,389 | - | NCBI |
| | | Cephalochordata | *Branchiostoma floridae* | ABEP02000017.1 | 29,201 | 29,618 | - | NCBI |
| | Echinodermata | Asteroidea | *Asterias amurensis* | GAVL01049438.1 | 1 | 372 | - | NCBI-TSA |
| | | Asteroidea | *Asterias rubens* | GAUU01010066.1 | 1 | 370 | + | NCBI-TSA |
| | | Asteroidea | *Asterias forbesi* | SRR1138708 | n/a | n/a | n/a | NCBI-SRA |
| | | Asteroidea | *Pisaster giganteus* | SRR11293188 | n/a | n/a | n/a | NCBI-SRA |
| | | Asteroidea | *Pisaster ochraceus* | SRR11293187, SRR10982254, SRR10982249 | n/a | n/a | n/a | NCBI-SRA |
| | | Asteroidea | *Patiria miniata* | AKZP01087997.1 | 1879 | 2303 | + | NCBI |
| | | Asteroidea | *Patiriella regularis* | CYSQ010112690.1 | 45 | 475 | - | NCBI |
| | | Asteroidea | *Acanthaster planci* | BDGF01006146.1 | 22,662 | 23,102 | + | NCBI |
| | | Asteroidea | *Echinaster spinulosus* | GAVE01110158.1 | 49 | 503 | - | NCBI-TSA |
| | | Ophiuroidea | *Ophionereis fasciata* | CZLG010265874.1 | 525 | 944 | + | NCBI |
| | Hemichordata | Enteropneusta | *Saccoglossus bromophenolosus** | No source data | n/a | n/a | n/a | n/a |
| | | Enteropneusta | *Saccoglossus kowalevskii** | NW_003116016.1 | 11,881 | 12,316 | - | NCBI |
| | | Enteropneusta | *Harrimaniidae sp. N. clade 3 JTC-2014* | SRR1695462 | n/a | n/a | n/a | NCBI-SRA |
| | | Enteropneusta | *Ptychodera flava* | BCFJ01016025.1 | 62,725 | 63,164 | + | NCBI |
| Protostomia | Brachiopoda | Lingulata | *Lingula anatina* | NW_013580819.1 | 173,755 | 174,151 | + | NCBI |
| | Phoronida | | *Phoronis australis* | NMRA01000149.1 | 753,300 | 753,694 | + | NCBI |
| | Annelida | Polychaeta | *Capitella teleta* | AMQN01027543.1 | 126 | 490 | + | NCBI |
| | | Polychaeta | *Arenicola marina* | SRR2005653 | n/a | n/a | n/a | NCBI-SRA |
| | | Polychaeta | *Hydroides elegans* | LQRL01164149.1 | 3,818 | 4,257 | - | NCBI |
| | | Clitellata | *Eisenia fetida* | CYRZ010654929.1 | 282 | 695 | + | NCBI |
| | | Clitellata | *Lumbricus rubellus* | scaffold682\|size41541 | 33,676 | 34,086 | - | DataSTORRE |
| | | Clitellata | *Olavius algarvensis* | SRR5421607 | n/a | n/a | n/a | NCBI-SRA |
| | | Clitellata | *Amynthas corticis* | FXXH010317067.1 | 93 | 484 | - | NCBI |
| | Mollusca | Gastropoda | *Pomacea diffusa** | No source data | n/a | n/a | n/a | n/a |
| | | Gastropoda | *Pomacea canaliculata* | PZQS01000002.1 | 30,270,917 | 30,271,327 | + | NCBI |

| | | | | | | | | |
|---|---|---|---|---|---|---|---|---|
| Protostomia | Mollusca | Gastropoda | *Conus tribblei* | LFLW010801771.1 | 6,342 | 6,711 | - | NCBI |
| | | Gastropoda | *Rapana venosa* | GDIA01241434.1 | 1 | 338 | - | NCBI-TSA |
| | | Gastropoda | *Littorina saxatilis* | Contig56765 | 8,751 | 9,136 | + | DRYAD |
| | | Gastropoda | *Haliotis discus hannai* | GIGJ01057632.1 | 465 | 866 | - | NCBI-TSA |
| | | Gastropoda | *Haliotis laevigata* | VKKT01005222.1 | 43,381 | 43,787 | - | NCBI |
| | | Gastropoda | *Haliotis tuberculata* | GEAU01287953.1 | 1 | 369 | + | NCBI-TSA |
| | | Gastropoda | *Elysia timida* | GBRM01107526.1 | 147 | 495 | + | NCBI-TSA |
| | | Gastropoda | *Elysia cornigera* | GBRW01213247.1, SRR1583663 | n/a | n/a | n/a | NCBI-TSA/SRA |
| | | Gastropoda | *Bathyberthella antarctica* | GFXA01051430.1 | 1 | 247 | - | NCBI-TSA |
| | | Bivalvia | *Mytilus galloprovincialis* | LNJA010333678.1 | 187 | 535 | + | NCBI |
| | | Bivalvia | *Bathymodiolus platifrons* | MJUT01034425.1 | 591,096 | 591,451 | + | NCBI |
| | | Bivalvia | *Modiolus philippinarum* | MJUU01052557.1 | 27,325 | 27,680 | - | NCBI |
| | | Bivalvia | *Pinctada fucata* | DRR050170 | n/a | n/a | n/a | NCBI-SRA |
| | | Bivalvia | *Pinctada martensii* | NIJJ01006429.1 | 9,898 | 10,264 | - | NCBI |
| | | Bivalvia | *Crassostrea gigas* | SZQM02000004.1 | 154,764 | 155,125 | + | NCBI |
| | | Bivalvia | *Crassostrea virginica** | KV918323.1 | 2,331,345 | 2,331,700 | - | NCBI |
| | | Bivalvia | *Nodipecten subnodosus* | GFNL01243408.1 | 1 | 252 | - | NCBI |
| | | Bivalvia | *Mizuhopecten yessoensis* | NEDP01005424.1 | 678,838 | 679,170 | + | NCBI |
| | | Aplacophora | *Alexandromenia crassa* | comp40353_c0_seq1 | 15 | 384 | + | DRYAD |
| Basal metazoa | Cnidaria | Anthozoa | *Acropora digitifera* | BACK02028654.1 | 1,815 | 2,284 | - | NCBI |
| | | Anthozoa | *Acropora cervicornis* | GASU01042147.1 | 1 | 361 | - | NCBI-TSA |
| | | Anthozoa | *Acropora hyacinthus* | GDIF01035769.1 | 16 | 409 | + | NCBI-TSA |
| | | Anthozoa | *Stylophora pistillata* | LSMT01000472.1 | 108,604 | 109,085 | + | NCBI |
| | | Anthozoa | *Seriatopora hystrix* | SRR2300678, SRR3144612, SRR3144610 | n/a | n/a | n/a | NCBI-SRA |
| | | Anthozoa | *Madracis auretenra* | comp285766_c0_seq1 | 1 | 424 | + | |
| | | Anthozoa | *Pseudodiploria strigosa* | HACD01239751.1 | 1 | 266 | + | NCBI-TSA |
| | | Anthozoa | *Favia lizardensis* | GDZU01016292.1 | 24 | 495 | - | NCBI-TSA |
| | | Anthozoa | *Orbicella faveolata* | MZGG01000725.1 | 580,730 | 581,215 | - | NCBI |
| | | Anthozoa | *Platygyra daedalea* | c38845_g1_i1 | 1 | 505 | + | PDTD |
| | | Anthozoa | *Montipora capitata* | RDEB01000170.1 | 373,653 | 374,130 | - | NCBI |
| | | Anthozoa | *Galaxea fascicularis* | GFAZ01037277.1 | 7 | 471 | - | NCBI-TSA |
| | | Anthozoa | *Montastraea cavernosa* | comp748712_c0_seq1 | 1 | 216 | + | DRYAD |
| | | Anthozoa | *Porites astreoides* | GEHP01404864.1 | 216 | 699 | - | NCBI-TSA |
| | | Anthozoa | *Ctenactis echinata* | GDZV01002545.1 | 1 | 285 | - | NCBI-TSA |
| | | Anthozoa | *Anthopleura elegantissima* | GBXJ01057767.1 | 34 | 437 | + | NCBI-TSA |
| | | Anthozoa | *Actinia tenebrosa* | SRR3207346 | n/a | n/a | n/a | NCBI-SRA |

| | | | | | | | | |
|---|---|---|---|---|---|---|---|---|
| Basal metazoa | Cnidaria | Anthozoa | *Corynactis australis* | GELM01043505.1 | 5 | 483 | + | NCBI-TSA |
| | | Anthozoa | *Ricordea yuma* | SRR3201251, SRR3201252, SRR3201253 | n/a | n/a | n/a | NCBI-SRA |
| | | Scyphozoa | *Aurelia aurita* | GBRG01038552.1 | 181 | 625 | - | NCBI-TSA |
| | | Scyphozoa | *Chrysaora fuscescens* | SRR3180892 | n/a | n/a | n/a | NCBI-SRA |
| | | Scyphozoa | *Rhopilema esculentum* | GEMS01118701.1 | 1 | 382 | - | NCBI-TSA |
| | | Scyphozoa | *Periphylla periphylla* | SRR1915828 | n/a | n/a | n/a | NCBI-SRA |
| | | Staurozoa | *Calvadosia cruxmelitensis* | HAHC01109237.1 | 3 | 350 | + | NCBI-TSA |
| | | Staurozoa | *Lucernaria quadricornis* | HAHD01078658.1 | 1 | 405 | + | NCBI-TSA |
| | | Hydrozoa | *Craspedacusta sowerbyi* | SRR923472 | n/a | n/a | n/a | NCBI-SRA |
| | Placozoa | | *Trichoplax adhaerens* | ABGP01000509.1 | 21,534 | 21,930 | + | NCBI |
| | Porifera | Demospongiae | *Amphimedon queenslandica* | ACUQ01004887.1 | 26,508 | 26,870 | + | NCBI |
| | | Calcarea | *Sycon ciliatum* | ERR592860, ERR592861, ERR592862, ERR592868 | n/a | n/a | n/a | NCBI-SRA |
| | | Homoscleromorpha | *Oscarella carmela* | scaffold411 | 49,661 | 50,069 | - | Compagen |

[a]: 5' end predicted based on multiple sequence alignment
[b]: 3' end of TR inferred as three nucleotides downstream of box ACA
[*]: TRs cloned in this study
NCBI: National center for Biotechnology Information – Genome Database, URL: www.ncbi.nlm.nih.gov/genome/
NCBI-TSA: National center for Biotechnology Information – Transcriptome Shotgun Assembly Sequence Database, URL : www.ncbi.nlm.nih.gov/genbank/tsa/
NCBI-SRA: National center for Biotechnology Information – Sequence Read Archive Database, URL : www.ncbi.nlm.nih.gov/sra/ (TRs identified using INFERNAL from assembled SRA reads or BLAST against raw reads)
DataSTORRE: Stirling Online Repository for Research Data – University of Stirling, URL: datastore.stir.ac.uk
DRYAD: Dryad digital repository, URL: datadryad.org
PDTD: *Platygyra daedalea* transcriptome database, URL: http://ib.oregonstate.edu/~meyere/DB/Pdae/search.php
Compagen: A comparative genomics platform for early branching metazoan, URL: http://www.compagen.org/index.html

A

Supplemental Fig. S1

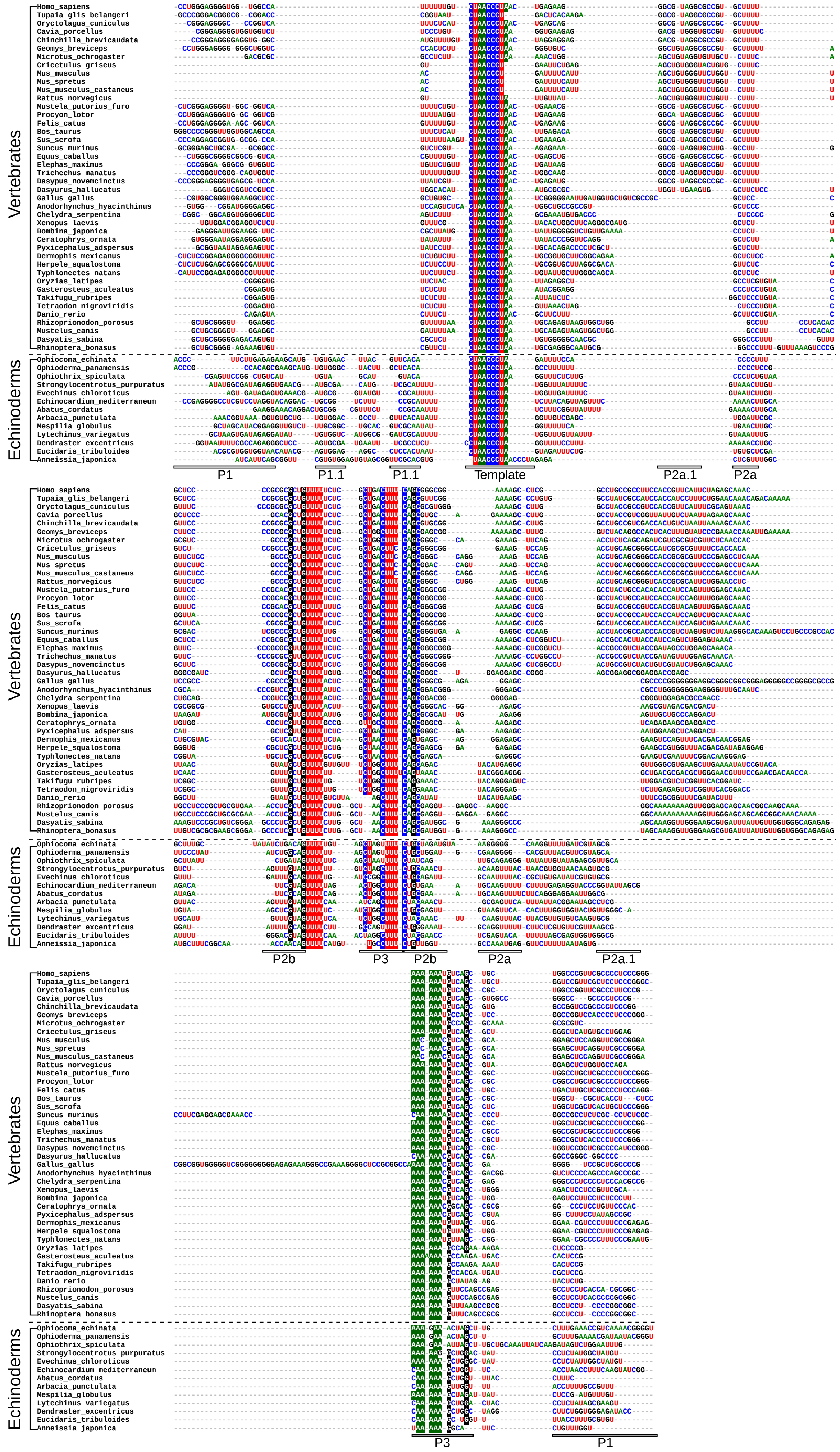

# B

# Supplemental Fig. S1

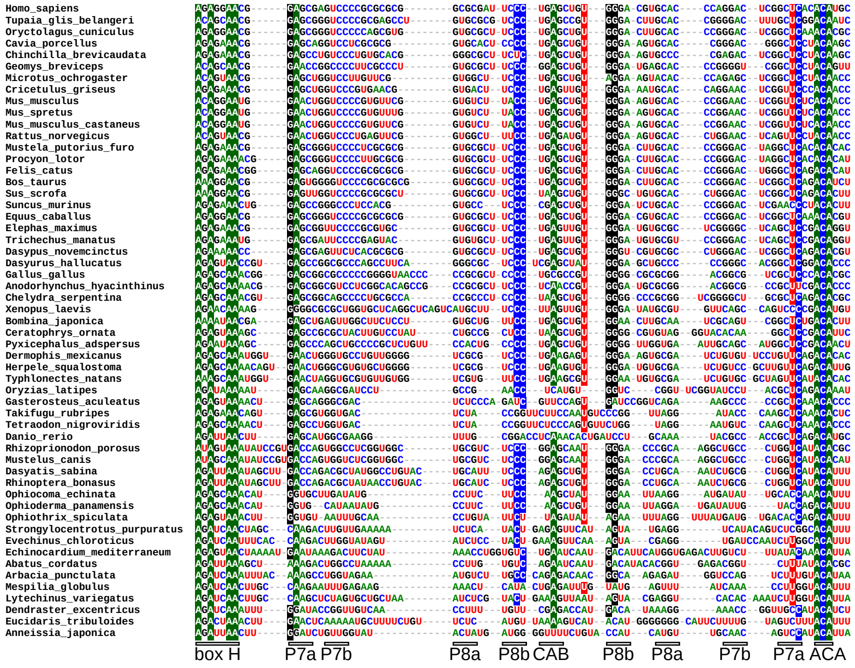

# Supplemental Fig. S2

## A

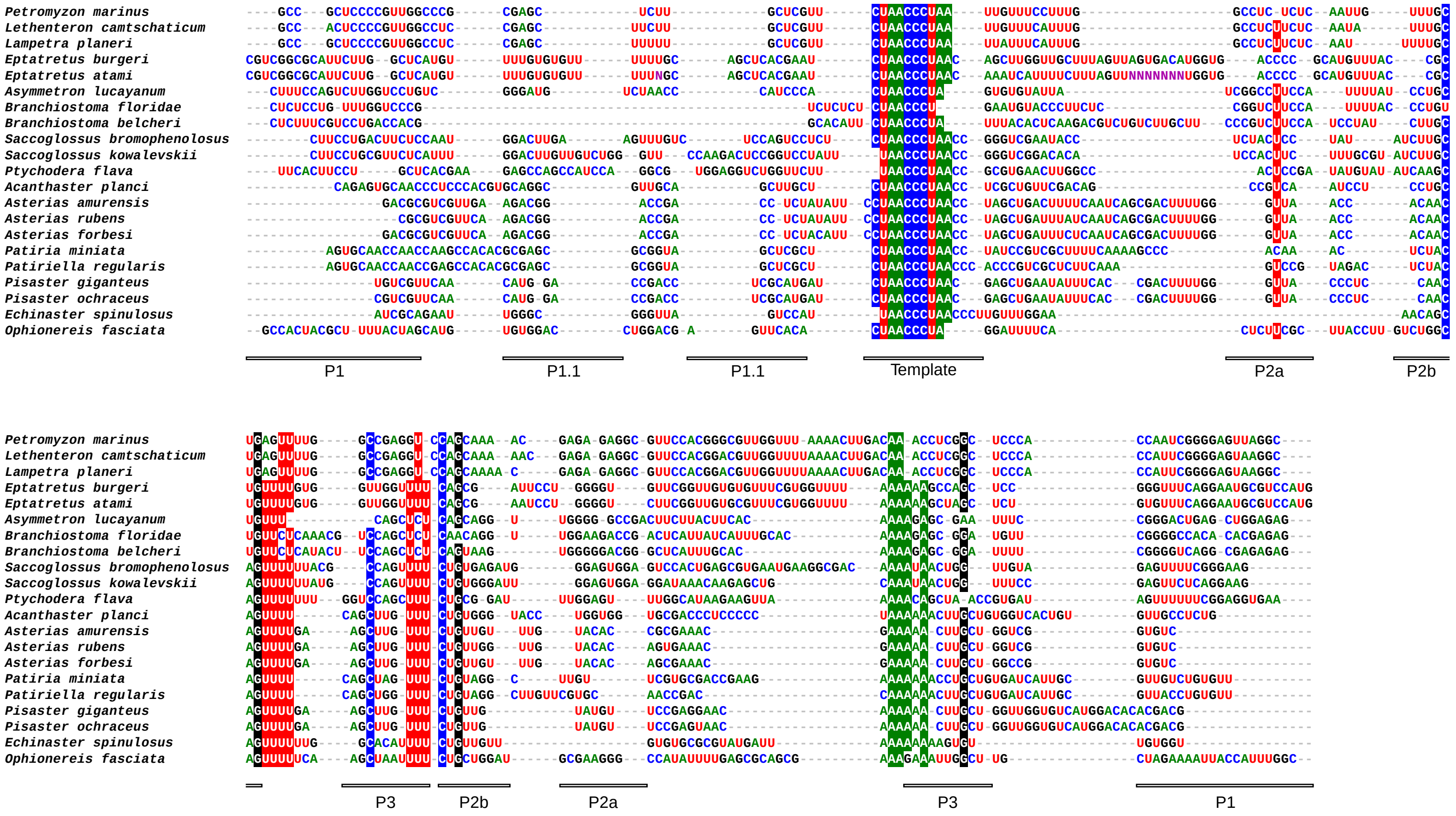


## B

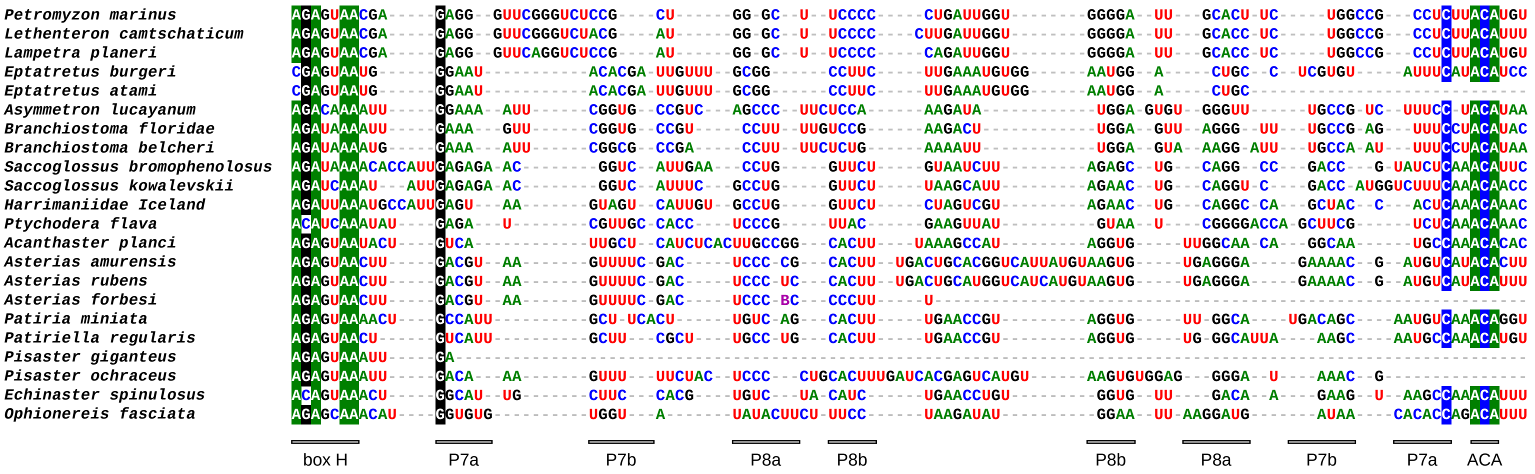

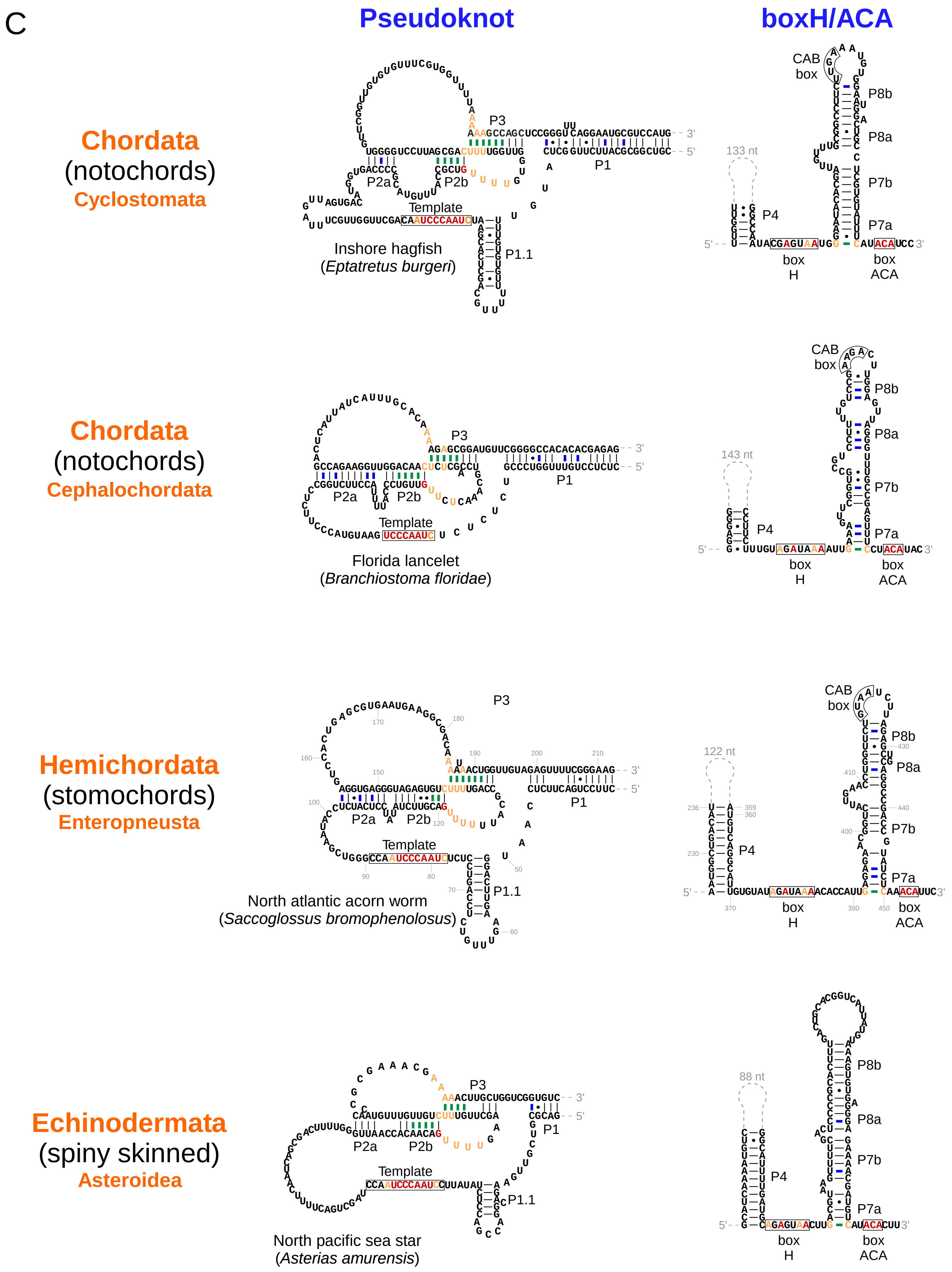
C
Pseudoknot
boxH/ACA
Chordata
(notochords)
Cyclostomata
P3
P2a
P2b
P1
Template
P1.1
Inshore hagfish
(Eptatretus burgeri)
CAB box
P8b
P8a
133 nt
P7b
P4
P7a
box H
box ACA
Chordata
(notochords)
Cephalochordata
Florida lancelet
(Branchiostoma floridae)
143 nt
Hemichordata
(stomochords)
Enteropneusta
North atlantic acorn worm
(Saccoglossus bromophenolosus)
122 nt
Echinodermata
(spiny skinned)
Asteroidea
North pacific sea star
(Asterias amurensis)
88 nt

# Supplemental Fig. S3

A

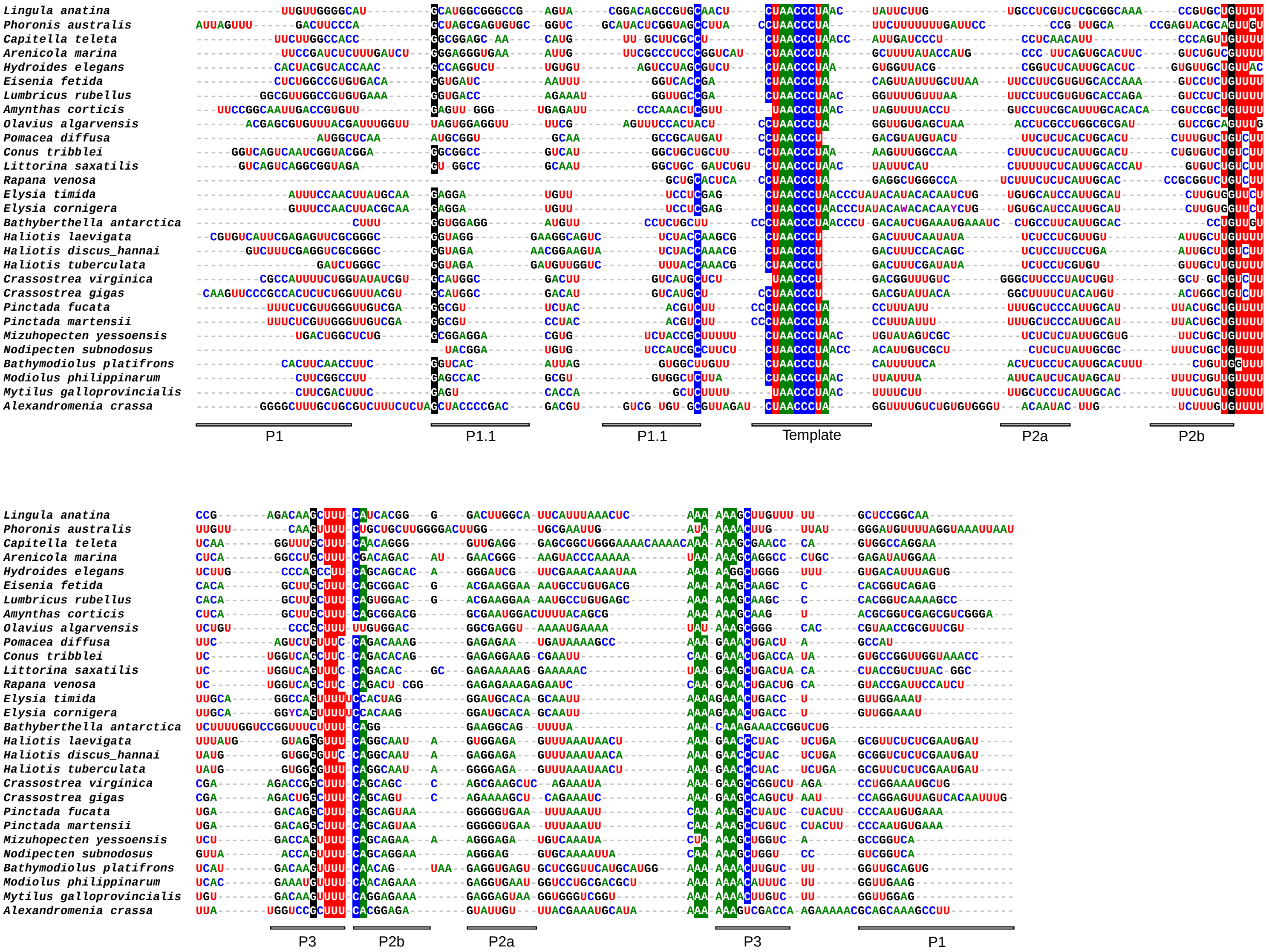


B

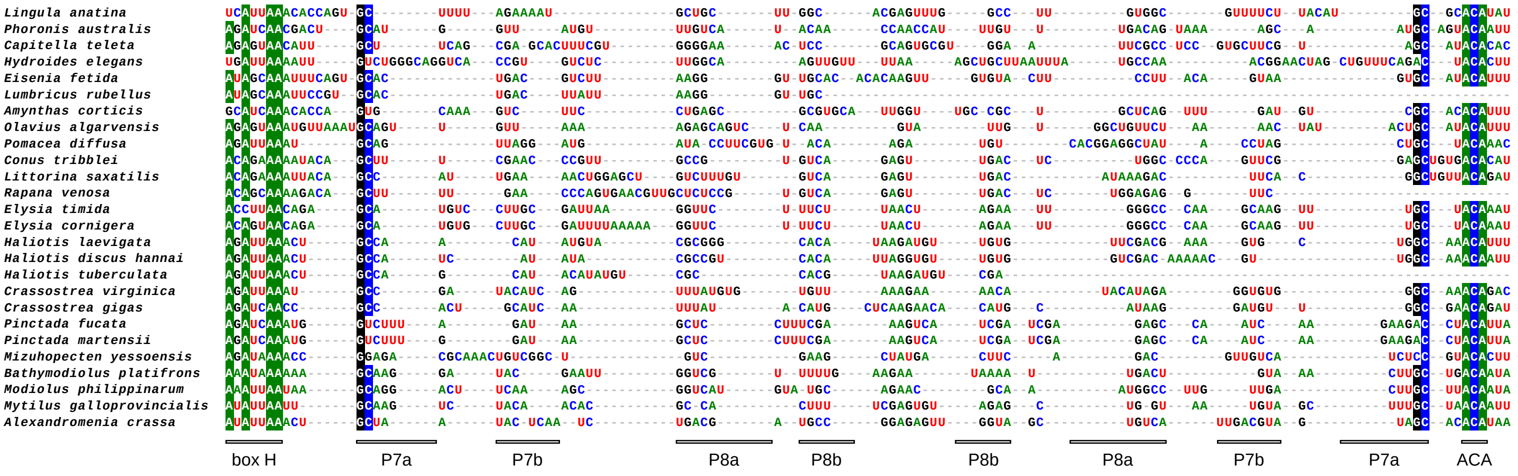

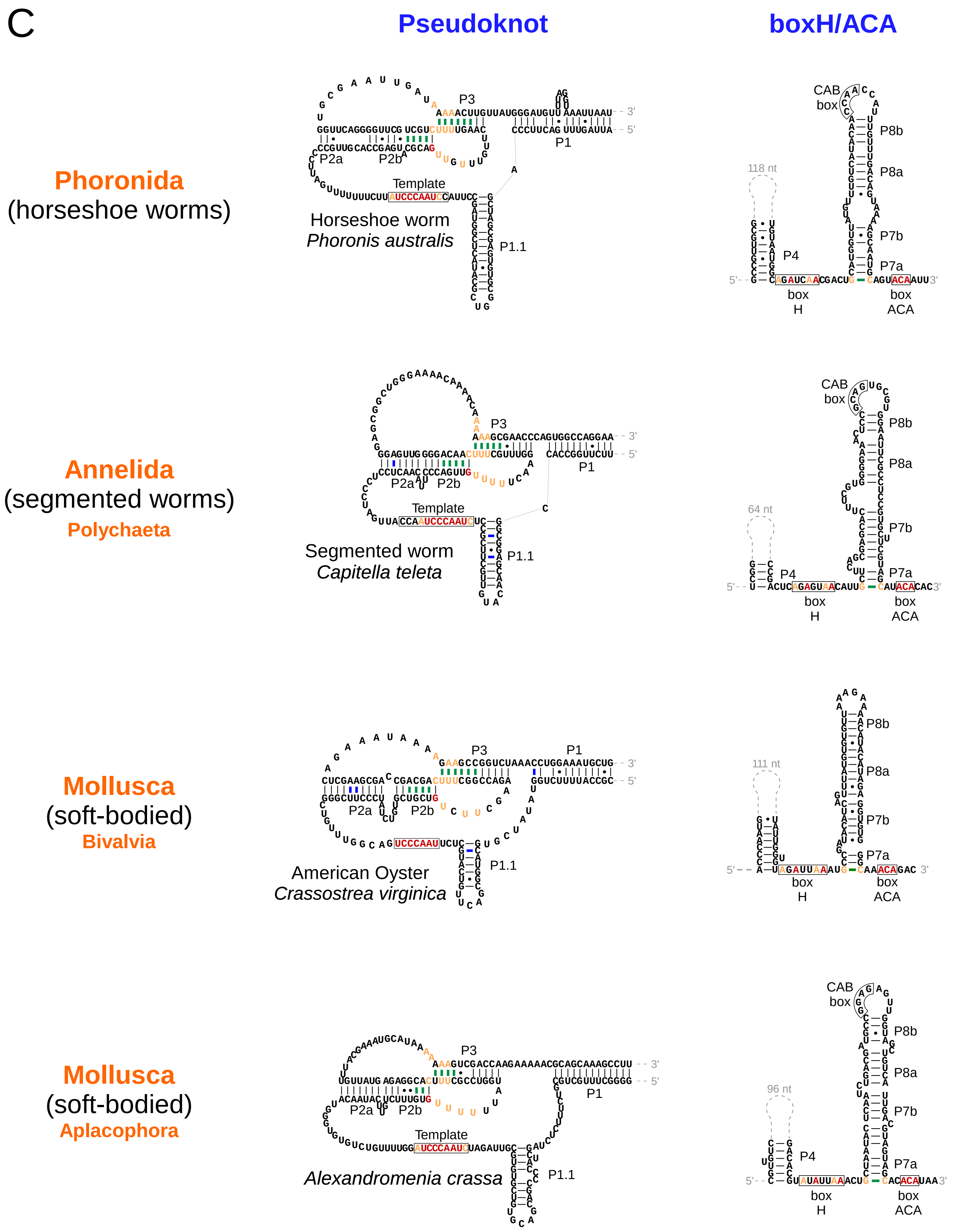
C
Pseudoknot
boxH/ACA
Phoronida
(horseshoe worms)
Horseshoe worm
Phoronis australis
Annelida
(segmented worms)
Polychaeta
Segmented worm
Capitella teleta
Mollusca
(soft-bodied)
Bivalvia
American Oyster
Crassostrea virginica
Mollusca
(soft-bodied)
Aplacophora
Alexandromenia crassa
Template
P3
P1
P2a
P2b
P1.1
CAB box
P8b
P8a
P7b
P7a
P4
box H
box ACA
118 nt
64 nt
111 nt
96 nt

# Supplemental Fig. S4

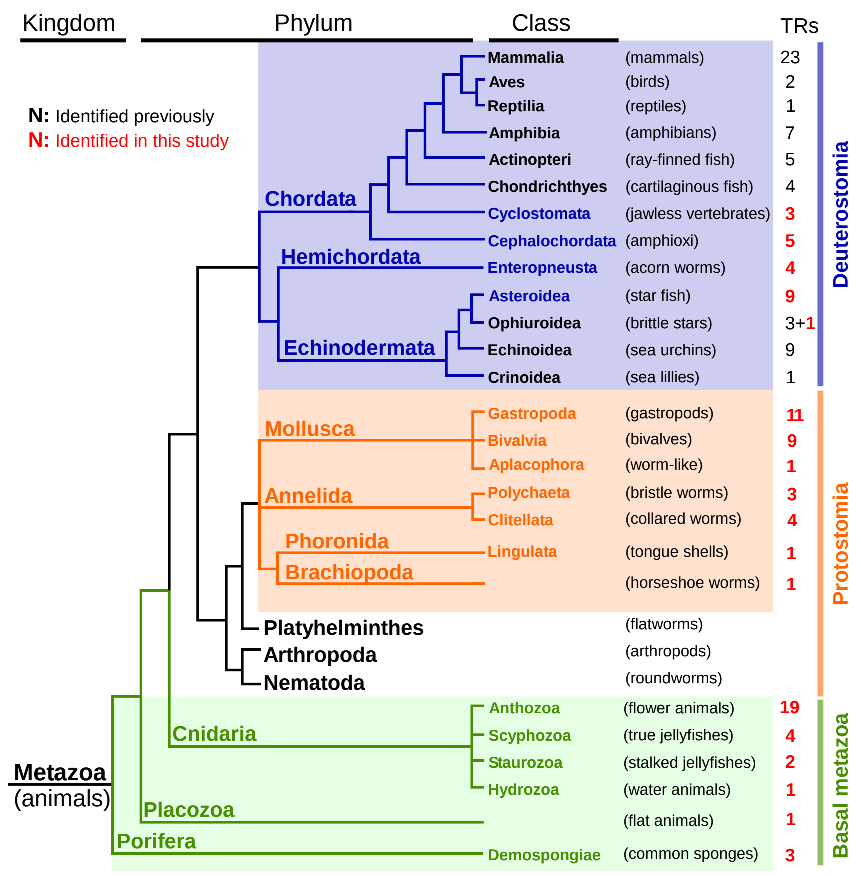

Supplemental Fig. S5

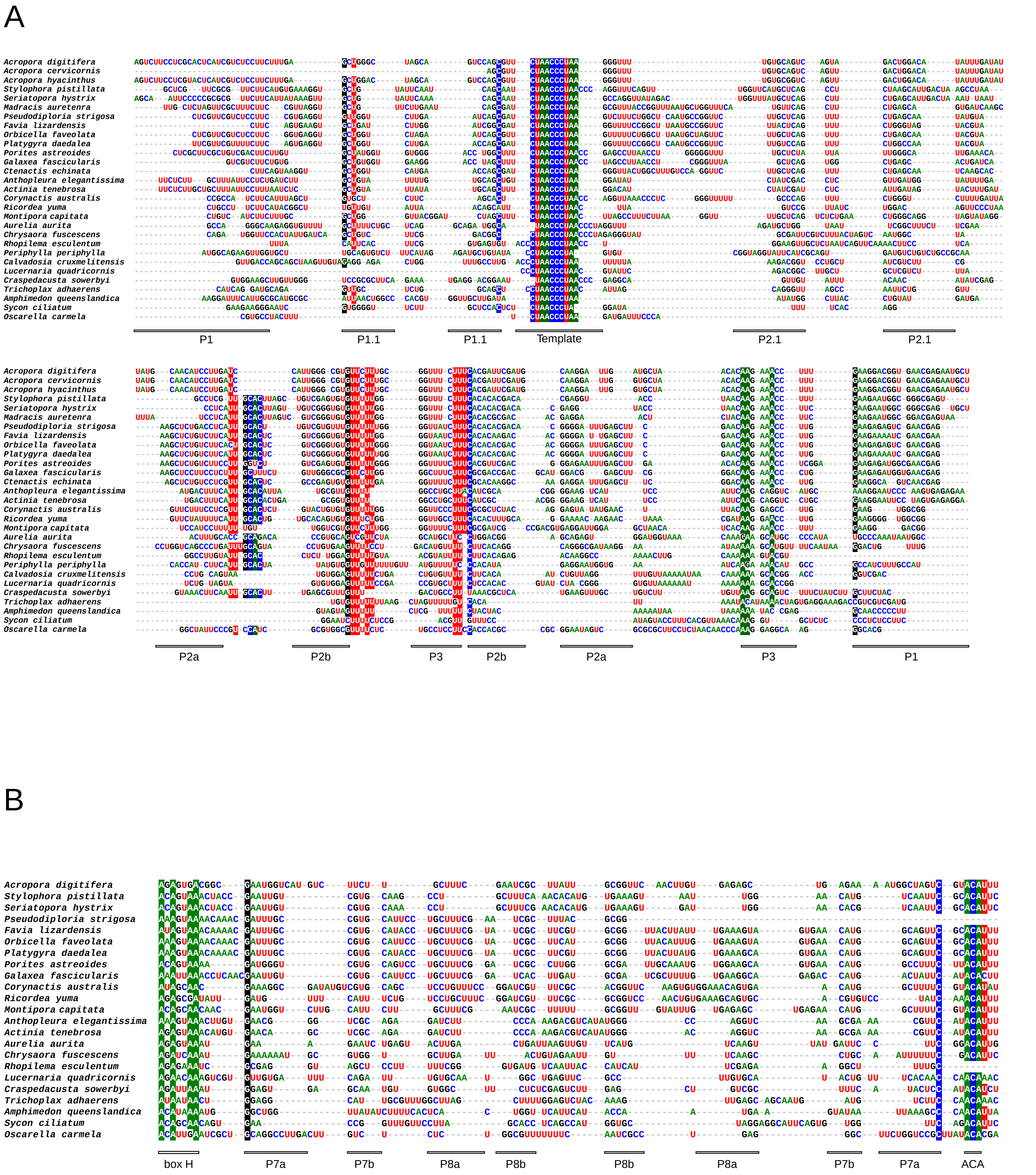

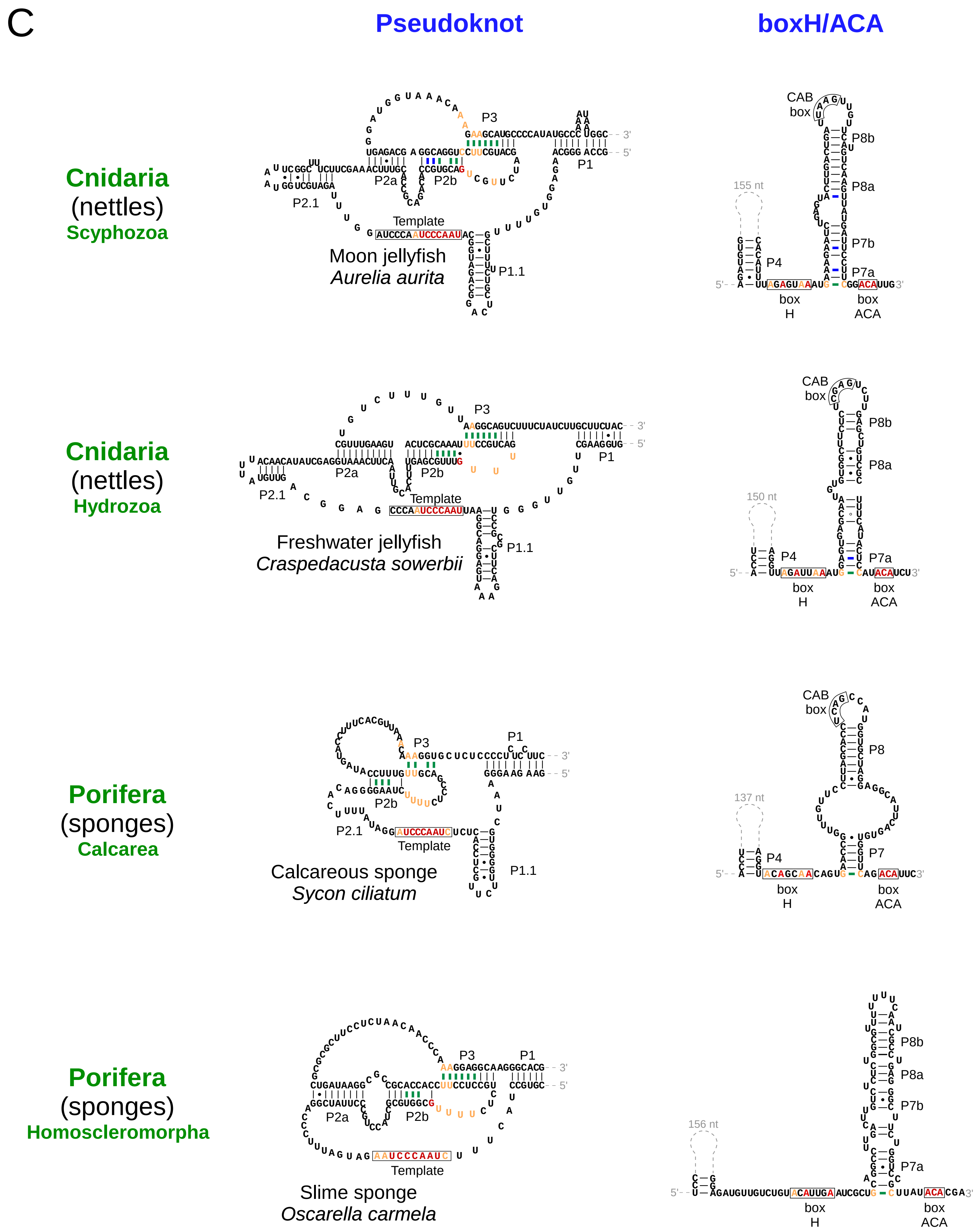
C
Pseudoknot
boxH/ACA
Cnidaria
(nettles)
Scyphozoa
Moon jellyfish
Aurelia aurita
Cnidaria
(nettles)
Hydrozoa
Freshwater jellyfish
Craspedacusta sowerbii
Porifera
(sponges)
Calcarea
Calcareous sponge
Sycon ciliatum
Porifera
(sponges)
Homoscleromorpha
Slime sponge
Oscarella carmela
P1
P1.1
P2a
P2b
P2.1
P3
P4
P7
P7a
P7b
P8
P8a
P8b
Template
CAB box
box H
box ACA
155 nt
150 nt
137 nt
156 nt
5'
3'

Supplemental Fig. S6

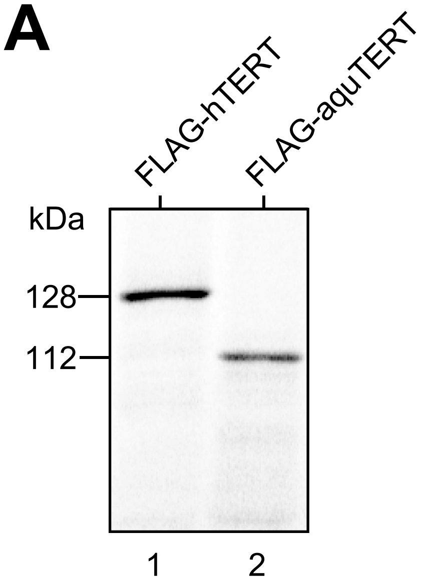


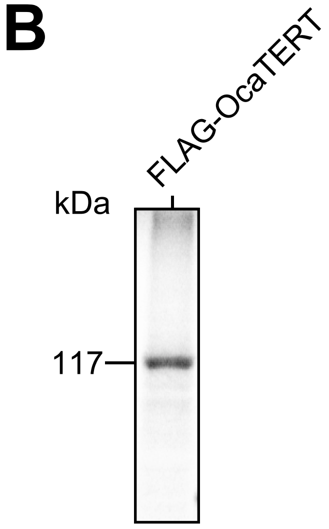

# Supplemental Fig. S7

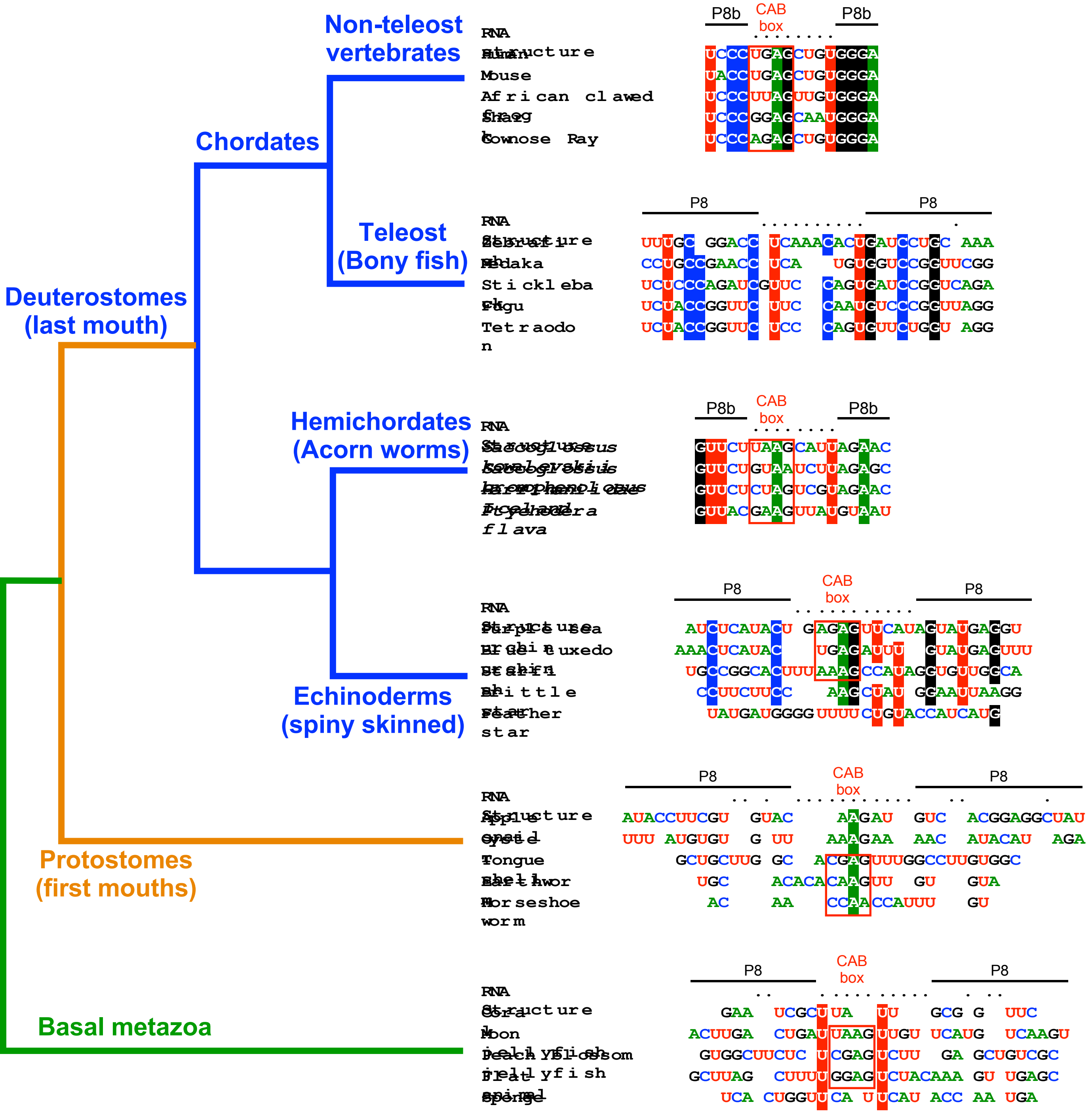

Supplement: msaa203_Supplementary_Data [file msaa203_Supplementary_Data.pdf]
